# Structures of the holoenzyme TglHI required for 3-thiaglutamate biosynthesis

**DOI:** 10.1016/j.str.2023.08.004

**Published:** 2023-08-30

**Authors:** Yanhui Zheng, Xiaoqing Xu, Xiaoli Fu, Xuerong Zhou, Chao Dou, Yue Yu, Weizhu Yan, Jingyuan Yang, Minqin Xiao, Wilfred A. van der Donk, Xiaofeng Zhu, Wei Cheng

**Affiliations:** 1 Division of Respiratory and Critical Care Medicine, State Key Laboratory of Biotherapy, West China Hospital of Sichuan University and Collaborative Innovation Center of Biotherapy, Chengdu, 610041, China; 2 Department of Neurology, Affiliated Hospital of North Sichuan Medical College, Institute of Neurological Diseases, North Sichuan Medical College, Nanchong, 637000, China; 3 Carl R. Woese Institute for Genomic Biology, University of Illinois at Urbana-Champaign, Urbana, IL 61801, USA.; 4 Department of Chemistry, University of Illinois at Urbana-Champaign, Urbana, IL 61801, USA.; 5 Howard Hughes Medical Institute, University of Illinois at Urbana-Champaign, Urbana, IL 61801, USA.; 6 Core Facilities of West China Hospital, Sichuan University, Chengdu, 610041, China; 7 College of Life Sciences, Sichuan University, Chengdu, 610065, China.; 8 These authors contributed equally to this work.; 9 Lead contact

**Keywords:** RiPP, X-ray, TglHI holoenzyme, Metalloenzymes, Mechanism

## Abstract

Structural diverse natural products like ribosomally synthesized and posttranslationally modified peptides (RiPPs) display a wide range of biological activities. Currently, the mechanism of an uncommon reaction step during the biosynthesis of 3-thiaglutamate (3-thiaGlu) is poorly understood. The removal of the *β*-carbon from the Cys in the TglA-Cys peptide catalyzed by the TglHI holoenzyme remains elusive. Here, we present three crystal structures of TglHI complexes with and without bound iron, which reveal that the catalytic pocket is formed by the interaction of TglH–TglI, and that its activation is conformation dependent. Biochemical assays suggest a minimum of two iron ions in the active cluster and we identify the position of a third iron site. Collectively, our study offers insights into the activation and catalysis mechanisms of the nonheme dioxygen-dependent holoenzyme TglHI. Additionally, it highlights the evolutionary and structural conservation in the DUF692 family of biosynthetic enzymes that produce diverse RiPPs.

## Introduction

Ribosomally synthesized and posttranslationally modified peptides (RiPPs) are a structurally diverse group of natural products with a wide array of biological activities.^1–5^ These molecules are derived from ribosomally synthesized precursor peptides that consist of an N-terminal leader peptide (LP) and a C-terminal core peptide (CP) required for recognition and posttranslational modification (PTM) by specific proteins, respectively. RiPPs share a similar biosynthetic process: the CP is modified by PTM enzymes at specific amino acids to create a mature product, and the LP is then removed.^6,7^ Groups of RiPPs have been characterized depending on different PTM processes, including macrocyclization (e.g., lanthipeptides, sactipeptides, ranthipeptides, thiopeptides, and streptides),^8–14^ backbone amide methylation,^15,16^ backbone epimerization,^17^ and backbone thioamide formation.^18,19^ The diverse precursors and PTM enzymes yield structurally and functionally diverse molecules, such as nisin A,^20^ gymnopeptide B,^21^ JBIR-140,^22^ pheganomycin,^23^ and methanobactins.^24–27^

Recently, a pathway to a unique RiPP, 3-thiaglutamate (3-thiaGlu), encoded in the genome of the plant pathogen *Pseudomonas syringae pv. maculicola* (*P. syringae*) ES4326, was reported.^28,29^ 3-ThiaGlu and its derivatives may interfere with glutamate receptor signaling in plants.^30^ The compound is biosynthesized by an unusual strategy in which an amino acid is appended to the C-terminal carboxylic acid of a ribosome-synthesized precursor peptide (the scaffold peptide) catalyzed by a peptide amino acyl-tRNA ligase (PEARL). The appended amino acid is then modified by PTM enzymes to form 3-thiaGlu, after proteolytic cleavage from the scaffold peptide. Unlike traditional RiPPs, 3-thiaGlu is not directly derived from ribosomal synthesis but is biosynthesized by posttranslational modifications of an appended non-gene-encoded amino acid on the scaffold peptide.

**Figure 1.**
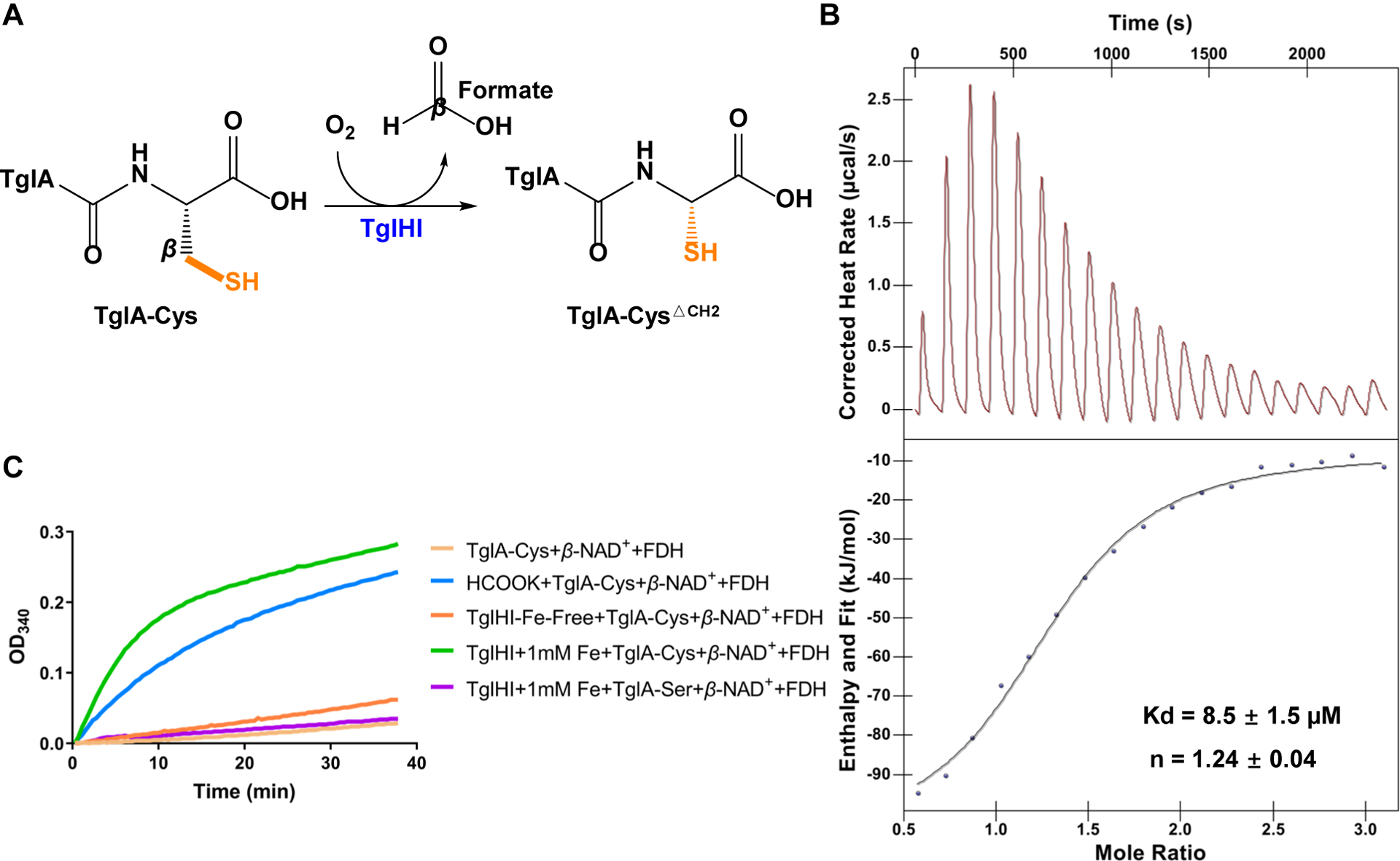
*In vitro* reconstitution of TglHI holoenzyme activity (A) The reaction catalyzed by TglHI. The TglHI complex catalyzes oxygen-dependent excision of the Cys *β* carbon, releasing C*β* as formate and generating TglA-Cys^ΔCH2^. (B) ITC measurement of the binding affinity between TglA-Cys and TglHI. The upper panel shows the original titration traces. (C) Formate detection of the minimum substrate analog of TglA-Cys modified by TglHI expressed in *E. coli* BL21 grown in M9 medium supplemented without (orange) and with (green) 1 mM ammonium ferrous sulfate in the presence of formate dehydrogenase (FDH) and *β*-NAD^+^. The reaction was monitored by UV-vis spectroscopy with continuous monitoring of absorbance at 340 nm, which corresponds to NADH produced by the hydrogen transfer executed by FDH from formate to *β*-NAD^+^. Low level background activity is seen in the absence of TglHI or when the C-terminal Cys of the substrate is mutated to Ser. This background activity results from spurious formate and is seen throughout the kinetic analyses in this study.

The *tgl* biosynthetic cluster of 3-thiaGlu consists of *tglA*, *tglB*, *tglH*, *tglI*, *tglF*, *tglE*, and *tglG* (Figure S1A). The PEARL enzyme TglB catalyzes the addition of a Cys from Cys-tRNA to the C-terminus of TglA to produce TglA-Cys (Figure S1B). The next unusual PTM reaction on TglA-Cys is performed by a complex formed by TglH and TglI, in which the cysteine *β*-methylene (*β*-CH_2_) of TglA-Cys is removed to generate TglA-Cys^ΔCH2^ (Figure 1A).^28^ A similar transformation was also reported recently in the biosynthesis of 3-thiahomoleucine, suggesting that carbon excision from Cys is used for the biosynthesis of a variety of 3-thia-α-amino acids.^31^ The molecular mechanism of TglHI-mediated carbon-excision from TglA-Cys remains unclear.

The TglHI complex is reminiscent of the recently reported holoenzyme, MbnBC,^32,33^ which generates an oxazolone and adjacent thioamide during the biosynthesis of methanobactin (Mbn, Figure S1C).^27,34^ MbnB is an iron-dependent enzyme from the DUF692 protein family, and MbnC acts as a recognition subunit for MbnA recruitment to the MbnBC holoenzyme. TglH shares only 16% amino acid sequence identity with MbnB (Figure S1D), and the TglH-catalyzed reaction is very different from that catalyzed by MbnB.^24,26,27,32^ TglI is a novel protein that has been predicted to contain a RiPP precursor peptide recognition element (RRE) domain,^28^ but lacks any structurally characterized homolog. Like MbnB and MbnC, TglH and TglI of the TglHI holoenzyme likely function as a catalytic and recruitment subunit, respectively, to achieve TglA-Cys modification.

The structure of the core biosynthetic machinery MbnBC in complex with the precursor substrate MbnA revealed new aspects of the mechanisms underlying substrate recognition and catalysis. Here, we report the structures of TglHI in the absence of Fe ions, with two Fe ions, and with three Fe ions (apoTglHI, TglHI-2Fe and TglHI-3Fe, respectively). The overall structure of TglH in TglHI resembles that of MbnB in MbnBC but several notable differences exist. Questions surrounding TglHI catalysis persist, including: How does TglHI recognize the C-terminal cysteine of the substrate TglA-Cys, which is much longer than MbnA? How does TglHI catalyze the reaction by removing the peptidyl-cysteine *β*-methylene group to form TglA-Cys^ΔCH2^? Does this enzyme require two or three Fe ions? And what are the chemical roles of these iron ions? In this study, we provide new insights into the molecular mechanism underlying the activation of and catalysis by the TglHI holoenzyme, whereby the catalytic center changes from an open cavity to a closed cavity upon the binding of Fe ions.

## Results

### *In vitro* reconstitution of TglHI activity

To investigate the mechanism and structure of the TglHI holoenzyme, TglH and TglI from *P. syringae* ES4326 were expressed in *Escherichia coli* (*E. coli*) BL21 (DE3) grown in Luria broth (LB) supplemented with 1 mM ammonium ferrous sulfate (see below). Sodium dodecyl sulfate–polyacrylamide gel electrophoresis (SDS-PAGE) analysis showed that TglH was expressed partially in soluble form, while TglI was insoluble when expressed alone and only soluble when co-expressed with TglH (Figure S2A). Additionally, a pull-down assay demonstrated the interaction of TglHI and the substrate TglA-Cys (Figure S2B), which was quantified by isothermal titration calorimetry (ITC), with a dissociation constant of 8.5 ± 1.5 μM (Figure 1B). To evaluate the activity of the TglHI with TglA-Cys, an *in vitro* enzymatic assay using ultraviolet‒visible (UV‒Vis) spectroscopy^28^ was optimized. Briefly, the absorbance at 340 nm was monitored to determine the presence of NADH generated by *β*-NAD^+^-dependent formate dehydrogenase (FDH) from *Candida boidinii* through hydride transfer from the second TglHI product formate to *β*-NAD^+^.^35^ As expected, a previously identified minimum substrate analog^29^ of TglA-Cys was modified by purified TglHI, as indicated by the increased level of detected NADH (Figure S2C). We also observed comparable NADH formation when using purified TglHI expressed in *E. coli* BL21 cultured in LB without supplementation with ammonium ferrous sulfate (Figure S2C). We conducted metal analysis on the two protein preparations using inductively coupled plasma‒optical emission spectrometry (ICP-OES) (Table S1), which showed about 1.50 ± 0.09 *equiv*. of Fe per TglHI for the supplemented sample and 0.91 ± 0.02 *equiv*. of Fe for the non-supplemented preparation. To gain a better understanding of the requirement of Fe ions, we then conducted experiments using a minimal nutrient M9 medium instead of LB medium. As anticipated, when using TglHI obtained from the M9 medium, NADH was not detectable above background (Figure 1C, Table S1), however, upon supplementing the M9 medium with 1 mM ammonium ferrous sulfate, a significant level of NADH formation was detected (Figure 1C). Additionally, no detectable NADH was observed above background when TglA-Ser was used as a substrate for this enzymatic assay (Figure 1C). We note that the background activity arises from low levels of formate in buffer^36^ resulting in background activity even when TglHI was omitted from the assay. Together with mass spectrometry data (*vide infra*), these results demonstrate that the purified TglHI removed the peptidyl-cysteine *β*-methylene group of TglA-Cys as formate *in vitro*, and that iron is essential for the TglHI enzymatic activity.

### Overall structure of apoTglHI

**Figure 2.**
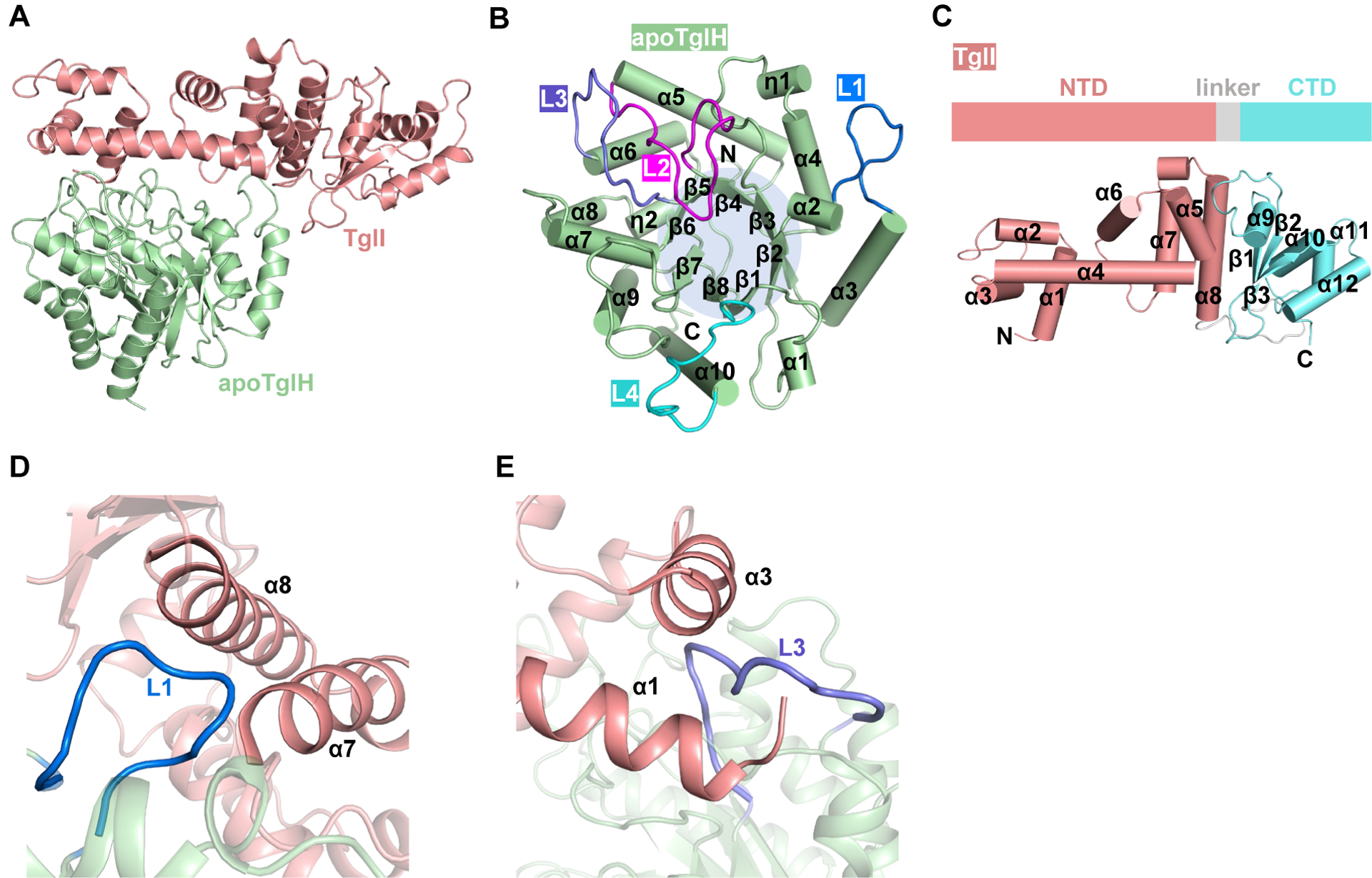
Overall structure of apoTglHI (A) Structure of apoTglHI. (B) The TIM-barrel structure of apoTglH. (C) Structures of TglI composed of the NTD and CTD. NTD, N-terminal domain; CTD, C-terminal domain. (D) Interaction between apoTglH and TglI by an extended loop (L1) of apoTglH inserting into a groove formed by the α7-α8 helixes of TglI. (E) Interaction between apoTglH and TglI involving another extended loop (L3) of apoTglH buried in grooves formed by the α1-α3 helixes of TglI.

To investigate the TglHI structure, we expressed TglHI in *E. coli* BL21 grown in LB without supplementation with ammonium ferrous sulfate and determined the TglHI structure by molecular replacement using the AlphaFold2-predicted structure model^37^ at approximately 3.39 Å resolution. Unlike the reported homologous structure of MbnB,^32,33^ no extra electron density in the active center of the TglH map was observed, suggesting that the crystallized protein lacks metal ions, and therefore, this structure was termed apoTglHI (Figure 2A, Table 1). ApoTglH adopts an eight-stranded triosephosphate isomerase (TIM)-barrel fold consisting of eight β-strands wrapped around ten α-helixes and two η-helixes (Figure 2B). This structure resembles those of other DUF692 family members, such as HsMbnB (PDB accession No. 3BWW), VcMbnB of the VcMbnABC complex (PDB accession No. 7DZ9),^32^ RrMbnB of the RrMbnABC complex (PDB accession No. 7FC0),^32^ and MtMbnB of the MtMbnBC complex (PDB accession No. 7TCX).^33^ TglI consists of an N-terminal helix domain (NTD), a linker loop, and a C-terminal domain (CTD) identified as an RRE domain (Figure 2C). The TglI CTD is composed of several helices and three β-strands for the binding of substrate TglA-Cys. A DALI server search returned no structural homologs to the NTD of TglI, indicating that it has a novel fold.

Although apoTglH and MbnB exhibit comparable overall structures, the specific inter-subunit interactions of apoTglHI are distinct from those of MbnBC. Two extended loops of apoTglH, L1 (residues 41–53) and L3 (residues 151–166), are inserted into two grooves formed by α7–8 (G2) and α1–3 (G1) of the TglI NTD, respectively, forming a mortise and tenon-like structure (Figures 2D and 2E). L1 embedded in G2 thereby forms a hydrophobic interface I with a buried surface area of ~573 Å^2^ (Figure S2D). Moreover, L3 buried in G1 forms a hydrophobic interface II with a buried surface area of ~320 Å^2^ (Figure S2E). These observed interfaces are different from those of MbnBC, in which the NTD of MbnC is sandwiched by two extended loops of MbnB, and a hairpin loop between the β2- and β3-sheets of MbnC inserts into the α1- and α2-helixes of MbnB (Figures S2F and S2G).^32^ Notably, the TglI CTD is not involved in the interaction between apoTglH and TglI, likely because of the role of the CTD in recognition of the longer LP of the cognate substrate.

To verify these structural observations, two TglH mutants were generated by deleting residues 42–50 in L1 (ΔL1) or residues 155–164 in L3 (ΔL3) and two TglI mutants were prepared by deleting the N-terminal residues 1–15 (Δα1) or an internal fragment α3 (Δα3) involved in forming G1. Co-expression of these TglHI variants demonstrated that the L1 and L3 truncations resulted in insoluble TglH (Figure S2H), and TglI^Δα1^ and TglI^Δα3^ abolished interaction with TglH. Furthermore, a pull-down assay demonstrates that the destruction of the CTD domain of TglI in TglHI resulted in the loss of interaction between TglA-Cys and TglHI (Figure S2I). These biochemical assays further support the structural observations and demonstrate that the identified regions are necessary for formation of the active TglHI holoenzyme. Taken together, the interaction interfaces may provide an explanation for the ability of TglHI to be copurified as a complex rather than a single protein.

### Formation of the catalytic cavity of TglHI upon Fe binding

As previously reported, TglH is a member of the DUF692 family and is an Fe-dependent enzyme that is believed to require at least one ferrous ion.^32,33^ This hypothesis was supported by the loss of TglHI activity with iron-free TglHI and with TglHI pretreated with H_2_O_2_ (Figures 1C and S3A, Table S1). However, treatment of EDTA merely attenuated the enzymatic activity of TglHI instead of abolishing the activity (Figure S3B).

To further explore the activation of TglHI, structures with iron bound to the active site were needed. Therefore, *E. coli* BL21 containing a *tglHI* plasmid were cultured in LB medium supplemented with 1 mM ammonium ferrous sulfate. This procedure resulted in TglHI crystallizing with iron and structures of TglHI with two or three Fe atoms were determined and named TglHI-2Fe (3.25 Å) and TglHI-3Fe (3.49 Å), respectively (Figures S3C and S3D). Noteworthy, the structure of TglHI-3Fe was obtained by co-expression with the substrate TglA-Cys and the addition of 1 mM ammonium ferrous sulfate to the medium. Regrettably, electron density for TglA-Cys was not observed in the map of TglHI, likely due to the poor solubility and flexibility of TglA-Cys. The structural models of TglHI-2Fe and TglHI-3Fe were supported by ICP‒OES, which illustrated that the number of Fe ions per heterodimer of TglHI-2Fe and TglHI-3Fe was 1.50 ± 0.09 and 2.48 ± 0.18, respectively (Table S1). Thus, co-expression of TglA-Cys increases the iron content of TglHI.

**Figure 3.**
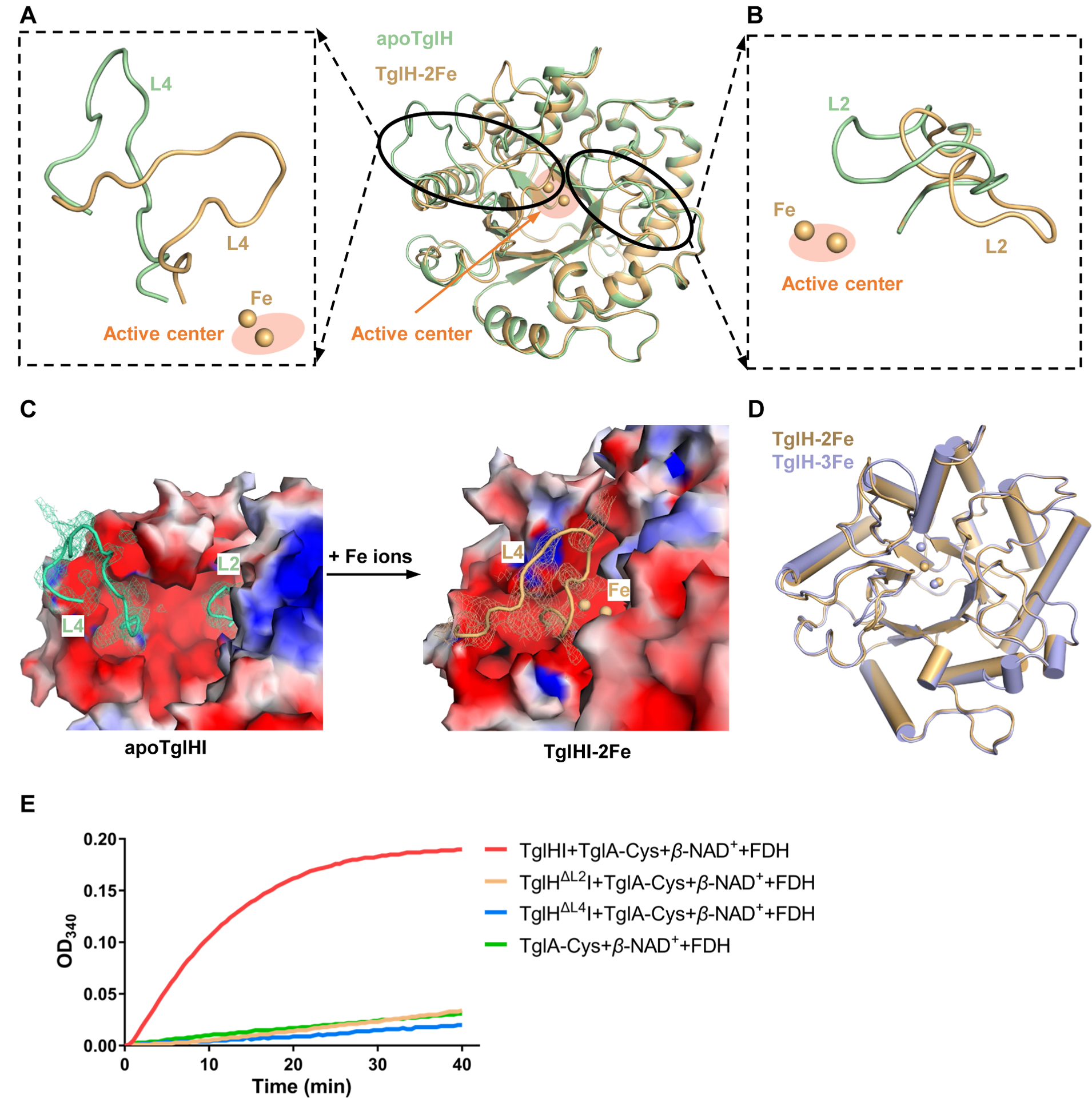
Formation of the catalytic cavity of TglHI induced by binding of Fe ions (A) Structural superposition of L4 in apoTglH and TglH-2Fe. Compared to apoTglH, the L4 in TglHI-2Fe closes above the active center. (B) Structural superposition of L2 in apoTglH and TglH-2Fe. Compared to apoTglH, L2, located below TglI, is far from the active center. (C) Formation of the closed conformation of TglHI. When iron is present, the two loops (L2/L4) of TglH undergo conformational changes to form a closed active site cavity. Electron density maps contoured with a 2Fo-Fc map at 0.8 σ for the L2/L4 loops. (D) Structural superposition of TglH-2Fe and TglH-3Fe. (E) *In vitro* activity of TglHI mutants (TglH^ΔL2^, TglH^ΔL4^) to modify the minimum substrate analog of TglA-Cys. These two deletion mutants remove the L2 and L4 structures of TglH, respectively.

Structural comparisons between TglHI without and with Fe ions demonstrated that the Fe-bound structure has undergone significant conformational changes in TglH (RMSD: 2.95 Å), but not TglI, within the TglHI complex (Figures 3A, 3B and S4A). Structural comparison between apoTglHI and TglHI-2Fe revealed that loops L4 (residues 258–275) and L2 (residues 107–126) of TglH are in very different orientations. Loop L4 closes over the active site in the TglHI-2Fe cap whereas loop L2 swings away from the active center (Figures 3A and 3B). The conformational changes in the loops induced by binding of two Fe ions result in the overall transition of TglH from an open to a closed active site cavity (Figure 3C). In addition, the changes upon iron binding enhance the hydrophobicity of the active center with an increase in buried surface area of about 309 Å^2^ (from ~493 Å^2^ to ~802 Å^2^) (Figures S4B and S4C). These structural observations emphasize the significance of L2 and L4 for the formation of a closed cavity upon binding of Fe ions. Structural superposition of TglHI-2Fe and TglHI-3Fe illustrated no significant conformational changes (RMSD: 0.63 Å) (Figure 3D), implying that the third iron plays a minor role in the TglHI-3Fe structure.

To verify the importance of L2 and L4 for TglHI activation, we generated two TglH mutants, ΔL2 (deleted residues: 110–120) and ΔL4 (deleted residues: 263–271). Enzymatic assays demonstrated that the two mutants exhibited TglHI activity equal to negative control groups without any impact on solubility of the TglHI complex (Figures 3E and S4D). Additionally, ICP-OES analysis revealed that the two mutants, TglH^ΔL2^I and TglH^ΔL4^I, contained 0.78 ± 0.01 and 1.11 ± 0.02 *equiv*. Fe, respectively (Table S2). The Fe content is slightly less than that of the wild-type TglHI (1.50 *equiv*. in TglHI-2Fe). These observations collectively indicate that the L2 and L4 loops play a critical role in forming the active center required for the catalytic reaction but that the loops do not greatly change the iron binding. Structural comparisons across members of the MbnB homologs show that these flexible loops are not involved in holoenzyme assembly for MbnB (Figure S4E), illustrating that activation of the TglHI holoenzyme resulting from loop movement and Fe ion binding is unique to TglHI.

### Active site of TglH

**Figure 4.**
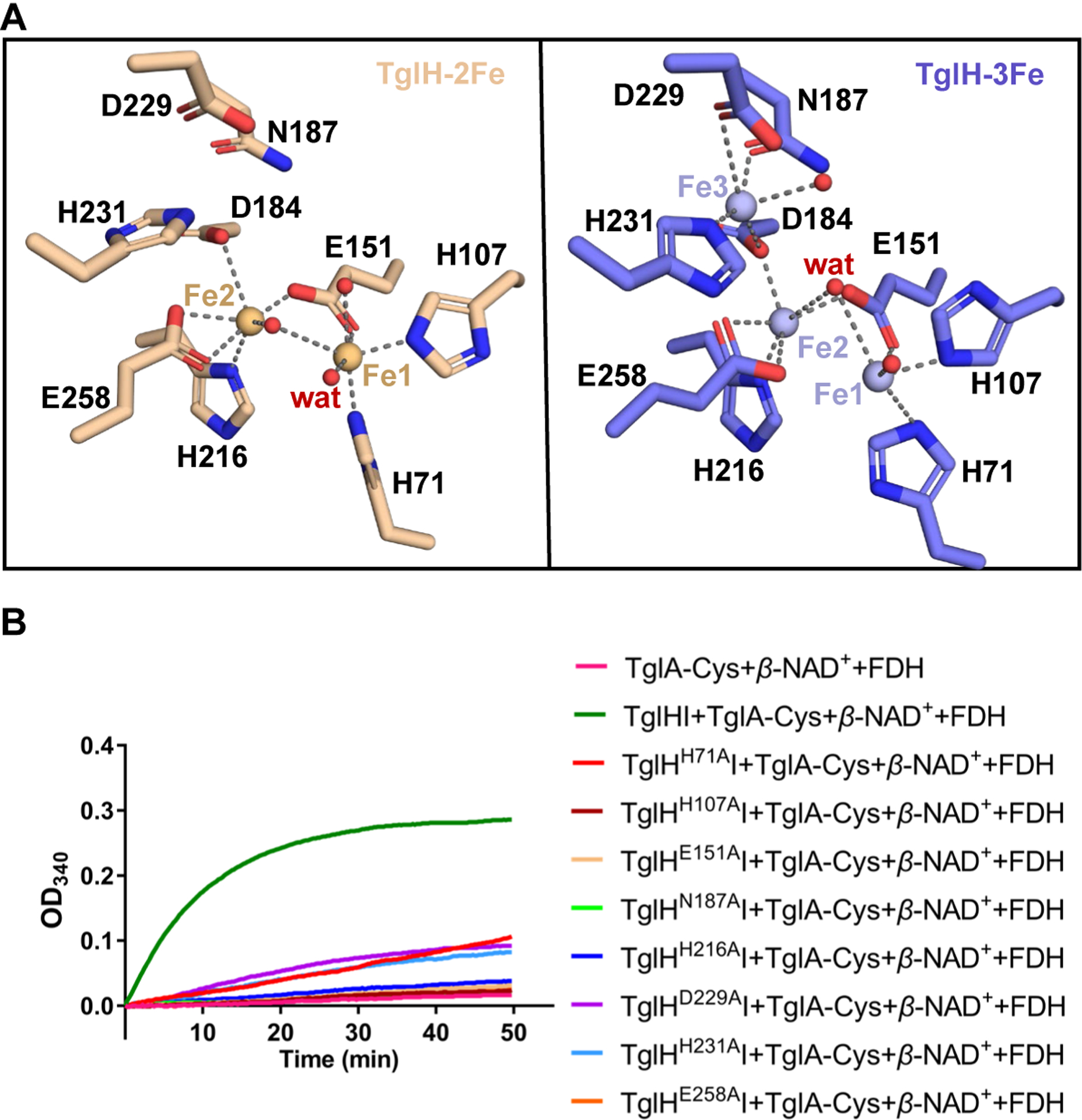
Active site of TglH (A) Interaction networks formed by the bi- or tri-iron cluster with surrounding residues in TglH. (B) *In vitro* activity of TglHI mutants involving residues engaged in Fe-coordination measured by the formate detection assay.

In the TglHI-2Fe and TglHI-3Fe structures, Fe_1_ and Fe_2_ are coordinated by His71, His107, Glu151, Asp184, His216, and Glu258, as well as water molecules (Figure 4A). The third iron (Fe_3_) in TglHI-3Fe is coordinated by Asp184, Asn187, Asp229, His231, and a water molecule. Two bidentate carboxylate interactions are formed by Glu151 to bridge Fe_1_ and Fe_2_ and by Asp184 to bridge Fe_2_ and Fe_3_ (Figure 4A). Structural superposition and sequence alignment suggest that the residues ligated to the Fe ions in TglH are highly conserved in MbnB homologs (Figure S1D). These observations suggest that the ability to form a tri-iron cluster in the active site is conserved across MbnB homologs. Notably, the electron density of Fe_3_ is weaker than that of Fe_1_ and Fe_2_ in the TglHI-3Fe map (Figures S3C and S3D), suggesting lower occupancy.

To investigate the importance of the residues involved in iron coordination, mutagenesis studies were carried out and the resulting TglHI variants were tested for activity using both the formate detection assay and matrix-assisted laser desorption ionization time-of-flight mass spectrometry (MALDI-TOF MS). The D184A mutation led to insoluble TglH (Figure S5A). The mutations H107A, E151A, N187A, H216A, and E258A resulted in TglHI activity that was akin to negative control experiments when analyzed by the formate detection assay with a negligible impact on the solubility of TglHI complexes (Figures 4B and S5B). These data suggest that these residues are important for activity. TglH-H71A, D229A and H231A showed low but clear enzymatic activity that was above background levels (Figure 4B). Two of these residues are ligands to Fe_3_ (Asp229 and His231) whereas His71 is a ligand to Fe_1_. Although the formate production activities of almost all mutants were very low, products with 14 Da mass decreases were detected by MALDI-TOF MS for a subset of the TglHI mutants when TglA-Cys was co-expressed in *E. coli* BL21. Exceptions were the H71A, E151A, E258A, and D184A (insoluble) variants, which did not produce *β*-carbon excision products (Figure S5C). The MALDI-TOF MS data were confirmed by HRMS ESI and MS/MS experiments after cleavage of the peptides with trypsin (Figures S5D and S5E). Taken together, these results imply that mutations H107A, N187A, H216A, D229A, and H231A greatly reduce activity of the purified enzymes but did not disrupt the Fe-cluster center sufficiently such that the carbon excision activity was entirely abolished. We therefore speculate that these residues may perhaps be rescued by other coordination groups, such as water molecules, to compensate for the lack of iron coordination, or that the ligand set reorients itself. Notably, mutations of Asp229 and His231 that are ligands to Fe_3_ still elicited nonnegligible activity both in the formate detection assay and MALDI-TOF MS analysis (Figures 4B and S5C). Furthermore, ICP-OES measurements showed that D229A and H231A variants (obtained by co-expression with TglA-Cys with 1 mM Fe supplementation) contained 1.32 ± 0.02 and 0.65 ± 0.01 *equiv*. Fe (Table S2), respectively, which is clearly less than the Fe content (about 2.48) of the TglHI-3Fe complex.

Conversely, variants H71A, E151A, and E258A, which all feature mutations to ligands of Fe_1_ or Fe_2_, did not show formation of product by MS. Of the mutants involving ligands to Fe_1_ or Fe_2_, only the H71A mutant showed formate production activity above background. This difference between *in vitro* assays with purified enzyme and the minimum substrate analog of TglA-Cys versus the co-expression experiment using full-length TglA-Cys may reflect the different experimental set-ups and possibly the concentrations of enzyme and substrate.

Collectively, the mutagenesis experiments demonstrate that most of the mutants only reduced the catalytic activities. In contrast, when the equivalent residues in MbnB were mutated, the catalytic activities of these variants were almost completely abolished^32,33^. However, for efficient formate excision, the assembly of the entire multinuclear irons cluster appears important as all mutants displayed greatly reduced formate formation activity (Figure 4B).

### Specific recognition of TglA-Cys by TglHI

Co-crystallization of TglHI with TglA-Cys variants was unsuccessful, and therefore, a structural model of TglHIA-Cys was generated using AlphaFold2 (named TglHIA-Cys-AF2)^37^ (Figure S6A). In this predicted model (which does not contain the Fe ions), the main features of the complex are similar, but TglH exhibits several differences compared with TglH in the crystal structures of apoTglH or TglH-2Fe, with the most noticeable difference adjacent to the Fe ions (Figure S6B). In contrast, TglI in the model is nearly identical to that in the experimental structures (Figure S6B). As shown in the TglHIA-Cys-AF2 structure (Figure S6C), residues 33–35 of TglA-Cys are predicted to be recognized by TglI with the formation of a β-hairpin loop formed by four interchain hydrogen bonds. This binding model is analogous to the recognition of the leader peptide of MbnA by MbnBC,^32^ indicating that the interaction pattern between the RiPP precursor peptide substrate and holoenzyme is conserved regardless of catalytic function. The model is also consistent with mutagenesis data on TglA-Cys and another AlphaFold model generated prior to the determination of the crystal structures of MbnBC and TglHI.^29^ In addition to the backbone interactions, several residues of TglI are predicted to contribute to the recognition of TglA-Cys with their side chains, including Arg183, Lys185, Tyr203, and Lys212 (Figure S6C). Supporting the AlphaFold2 model of substrate engagement, mutagenic replacement of these residues with Ala indicated that the activity of mutants of these residues decreased to varying degrees using the formate production assay (Figure S6D). The model also explains why 19 amino acids (residues 33–51) is the shortest fragment of TglA-Cys for activity by TglHI, as reported previously.^29^

**Figure 5.**
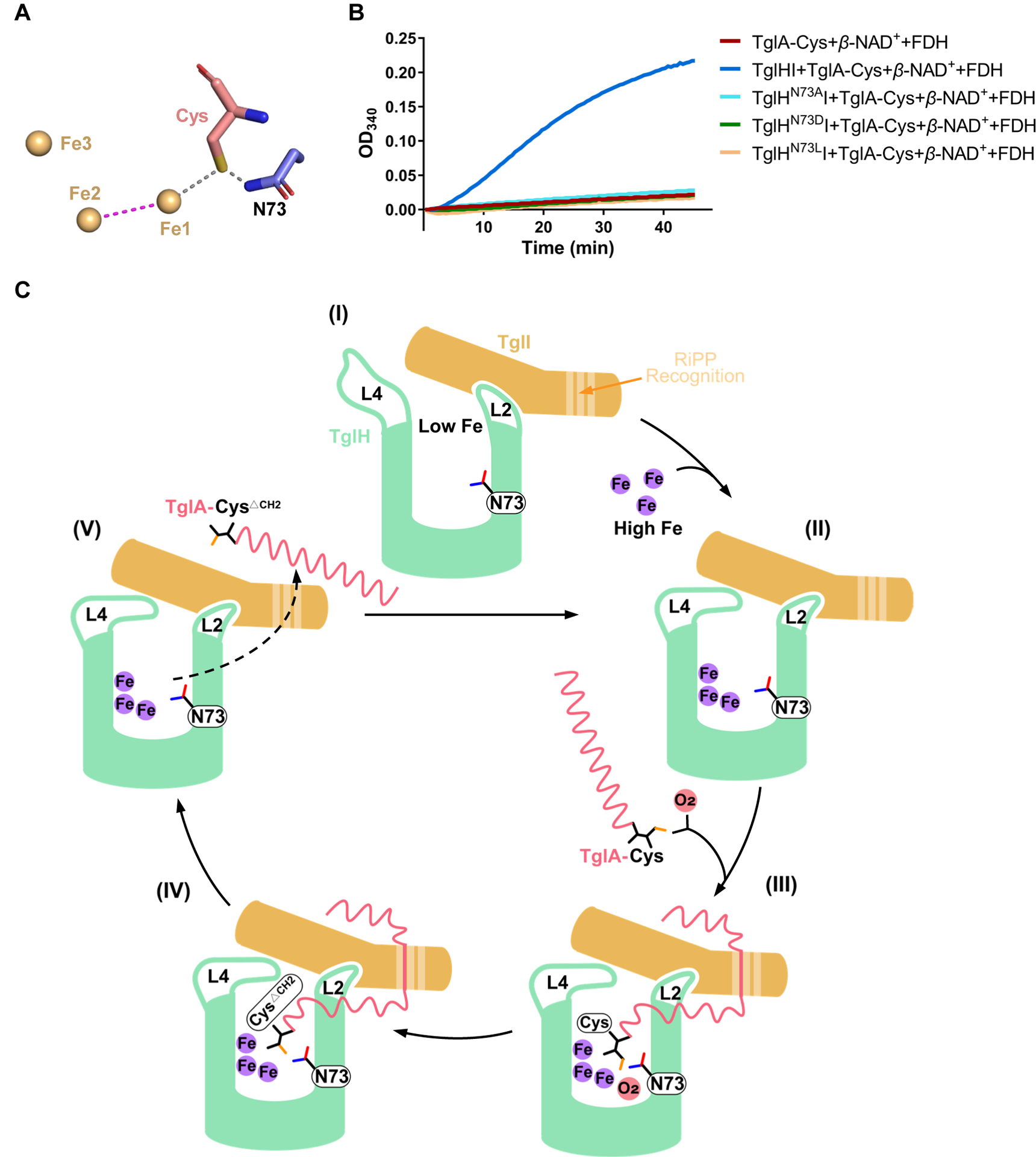
Proposed catalytic cycle of TglHI (A) Zoom-in of the structure of TglH with the C-terminal Cys of TglA modeled by comparison with the structures of VcMbnB and VcMbnABC (PDB accession No. 7DZ9). The AlphaFold2 model positions the side chain amide of Asn73 near the sulfur of Cys. (B) *In vitro* activity of TglHI variants (TglH^N73A^I, TglH^N73D^I and TglH^N73L^I) to modify the minimum substrate analog of TglA-Cys as measured by the formate production assay. (C) The TglA-Cys modification process mediated by TglHI. Asn73 of TglH is suggested to orient the Cys ligated to Fe1 for hydrogen atom abstraction by the peroxo intermediate through formation of a hydrogen bond between the thiolate of Cys and the amide −NH_2_ of Asn73. See Figure S6F for a detailed chemical description.

Interactions between VcMbnA-Cys18 and VcMbnB-Fe_1_ or between RrMbnA-Cys25 and RrMbnB-Fe_1_ occur by coordination of Cys to Fe_1_ of MbnB.^32^ Based on the structural superposition of TglHIA-Cys-AF2 and MbnABC, the C-terminal Cys of TglA-Cys is also predicted to directly ligate to the Fe_1_ of TglH (Figure 5A). To investigate the importance of the C-terminal Cys in TglHI catalysis, cysteine was substituted by serine, valine, and threonine (Figure S6E). None of these mutants were modified by TglHI based on the formate production assay, indicating the requirement of the sulfhydryl group for catalysis.

### Identification of a key catalytic residue

Given the structural similarity of TglH and MbnB, we analyzed the TglHI structure for residues that would be analogous to the proposed catalytic residues Asp240/242 of MbnB.^32^ No similar residue was identified; however, the side chain N-H of Asn73 in TglH was found to form a hydrogen bond with the thiolate of the C-terminal Cys of TglA-Cys in the AlphaFold2 complex (Figure 5A). To investigate the potential importance of this interaction for catalytic activity, Asn73 was substituted with Ala, Leu, or Asp. Activity assays demonstrated that the resulting variant proteins displayed equivalent behavior as the negative control experiments (Figure 5B), indicating that the presence of the side chain amide of Asn73 is essential for catalysis. Sequence-based alignment indicates that Asn73 is present only in TglH and its orthologs and not in MbnB (Figure S1D).

## Discussion

In the current study, we report several crystal structures of TglHI from *P. syringae* ES4326 in the absence and presence of iron ions. Our structural and biochemical data provide new insights into the mechanisms of holoenzyme TglHI activation and catalysis of peptidyl (*S*)-2-mercaptoglycine biosynthesis. The overall structure of TglH resembles that of MbnB, demonstrating structural conservation of the core biosynthetic machinery for the RiPPs Mbn and 3-thiaGlu. TglH and MbnB both adopt a TIM barrel structure, with β-sheets wrapped around helices, forming stable complexes with TglI and MbnC, respectively. Surprisingly, clear differences in complex assembly are observed through structural comparison of TglHI and MbnBC. Two loops (L1, L3) of TglH are inserted into TglI, similar to a mortise-tenon joint structure rather than the structure formed by MbnC sandwiched between MbnB (Figures 2D, 2E, S2F and S2G). Moreover, the CTD of TglI is not involved in the assembly of TglHI in contrast to the β-hairpin loop of MbnC, which makes contacts with MbnB. Our data reveal that the DUF692 family proteins likely employ diverse assembly patterns with their substrate-engaging protein partners to produce diverse RiPPs, which is further exemplified by a recent DUF692 enzyme with a partner protein that is a transmembrane protein.^38^ Additionally, based on structure prediction and alignment, structurally similar assemblies to TglHI were found in numerous pathogenic bacteria, while their specific functions are still to be investigated.

The interaction with TglI is required for TglH catalytic activity, indicating that the TglHI complex acts as a holoenzyme for 3-thiaGlu generation, akin to MbnBC. The central cavity formed by TglH and TglI is conserved throughout the currently characterized members of the DUF692 family. Surprisingly, two extended loops (L2 and L4) from TglH are involved in the formation of this central cavity. In the closed state, two iron ions are bound in the active site (TglHI-2Fe), whereas in the open state, iron ions are absent from the active site (apoTglHI) (Figure 3C), indicating that the intrinsic flexibility of L2 and L4 allows Fe loading in TglH to result in TglHI activation. These loops exist uniquely within TglH as L2 is replaced by a hairpin loop in MbnB and L4 is absent from MbnB (Figure S4E). This difference could contribute to the production of diverse RiPPs by cognate biosynthetic machinery that otherwise have high structural conservation.

For example, all three irons present in the central cavity of the holoenzymes VcMbnBC and RrMbnBC are likely involved in catalysis, while another homolog, MtMbnBC, has been reported to require only two Fe ions for enzymatic activity.^32,33^ In this work, it appears that two Fe ions are sufficient for TglA-Cys modification. Most alanine mutants of the Fe ligands of TglHI have diminished enzymatic activity except for E151A, D184A (insoluble) and E258A (Figures 4B and S5C), rather than completely abolishing the activity as in the case of MbnBC.^32,33^ The alanine mutations associated with the third iron that resulted in soluble protein maintained enzymatic activity, based on MALDI-TOF MS analysis although the formate production assay show significant reductions in activity (Figures 4B and S5C). Thus, the observations of product, in conjunctions with TglHI structures (Figures 3 and 4A) and ICP-OES assays (Tables S1 and S2), suggest that the third iron may play a non-essential role.

Another key difference between TglHI and MbnBC substrates is the site of modification of their substrate peptides. TglA-Cys is modified by TglHI at the C-terminal cysteine, whereas the substrate MbnA of MbnBC is modified at two internal cysteines of the core peptide.^32^ Moreover, our data reveal potential additional factors that may contribute to the different catalytic mechanisms between TglH and MbnB. Asp240/242 have been proposed to act as a general base for the catalytic reaction of MbnB,^32^ but these residues are missing in TglH. Conversely, our data suggests that Asn73 of TglH is necessary for catalysis, whereby the amide NH_2_ of Asn73 may form a hydrogen bond with the Cys S*γ* (Figure S6F), and perhaps contribute to the proposed hydrogen atom abstraction^32^ from C*β* on Cys.

Collectively, based on the data in this and previous studies we suggest a potential catalytic cylce for the process facilitated by TglHI (Figure 5C). The reaction on TglA-Cys has some similarities to that catalyzed by HEPD,^39^ a dioxygen-dependent non-heme-iron dioxygenase. HEPD employs one iron as its sole cofactor to activate O_2_ and then cleaves a C-C bond in 2-hydroxyethylphosphonate to produce hydroxymethylphosphonate (HMP) and formate. Based on these prior findings,^28^ a catalytic mechanism for the TglHI-catalyzed process was formulated (Figure S6F). Similar to other non-heme-iron enzymes,^32,40–42^ the TglHI-catalyzed TglA-Cys modification is speculated to start with the activation of oxygen by Fe_1_^II^ ligated by the C-terminal cysteine (**1→2**). The generated Fe_1_^III^-O_2_^−^ superoxo species may perform H-atom abstraction (H-abs) from the C*β* of Cys to generate an Fe_1_^III^-hydroperoxo species and a C*β* radical (**2→3**). The cysteine of TglA-Cys may be oriented by Asn73 to facilitate the hydrogen abstraction. Subsequently, the C*β* radical may react with the Fe_1_^III^-hydroperoxide to generate a thioacetal and a Fe_1_^IV^=O complex (ferryl) (**3→4**). Alternatively, conversion from **3** to **4** may involve electron transfer from the C*β* radical to Fe_1_^III^, resulting in the generation of a Fe_1_^II^-hydroperoxo and a thioaldehyde, similar to the proposed mechanism for HEPD.^39^ The Fe_1_^II^-hydroperoxo might then form an Fe_1_^IV^-oxo and the liberated hydroxide can attack the thioaldehyde to arrive at the same species **4**.

Regardless of the details of its formation, the ferryl could then oxidize the thiohemiacetal by a proton-coupled electron transfer process to generate an oxygen-based radical, leading to C-C bond breakage to form the captodatively stabilized Gly radical and thioformate liganded to the Fe_1_ (**4→5→6**). This process would be analogous to the reaction catalyzed by HEPD.^39^ Next, a radical recombination with the thioformate ligand is proposed to result in the formation of the C*α* carbon-sulfur bond, akin to the formation of a thioether by isopenicillin N synthase.^42^ Finally, hydrolysis of the thioformyl group would result in the formation of formate (**6→7→8**).

**Figure 6.**
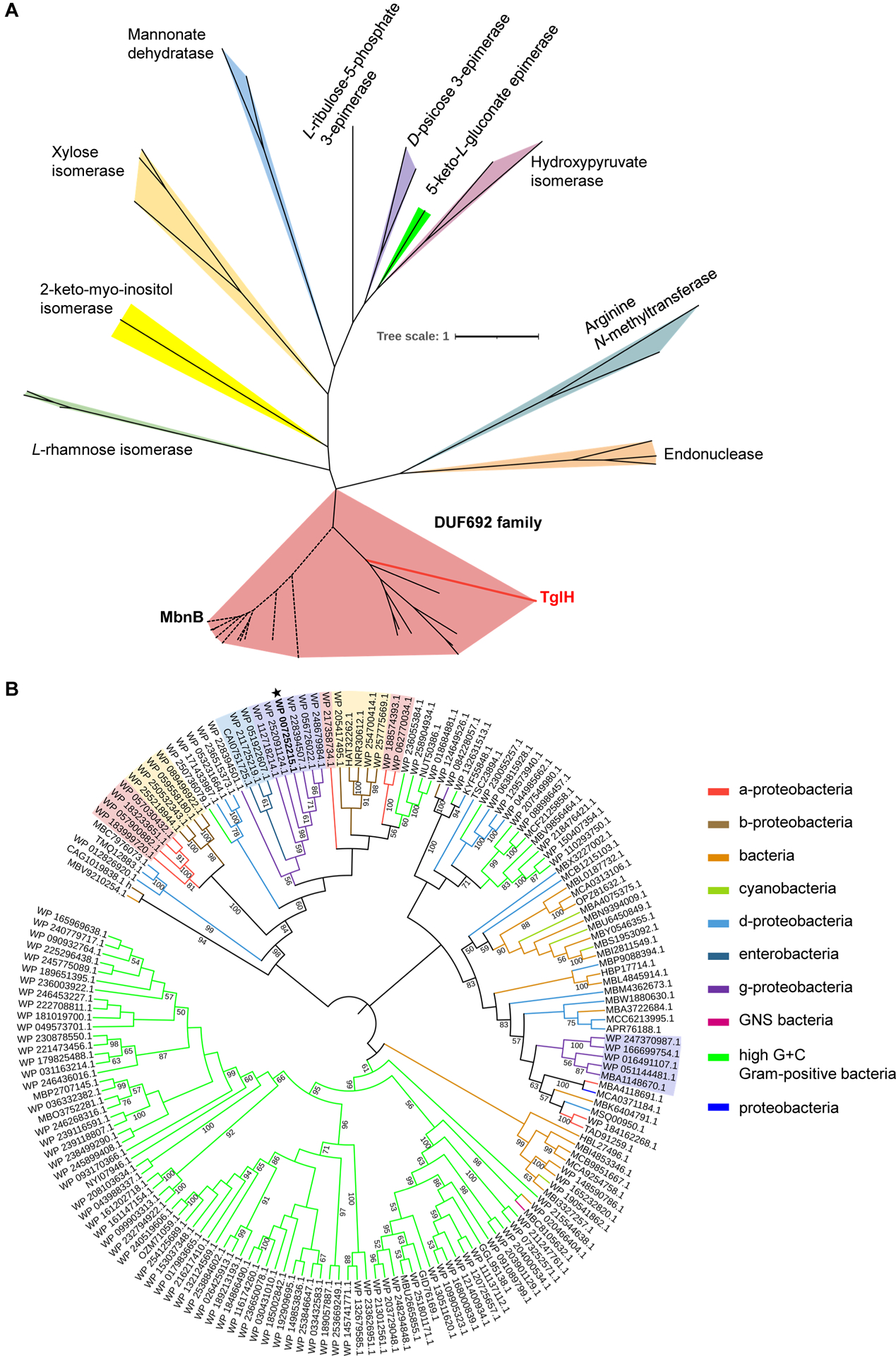
phylogenetic tree analysis of TIM-barrel-related enzymes and TglH homologues (A) Maximum likelihood tree based on representative protein sequences of TIM-related enzymes. TglH is highlighted in red font. TIM-related enzymes were searched in the EMBL-EBI database. (B) Maximum likelihood tree based on the protein sequences of TglH homologues. TglH is labeled with a black pentagram. Homologs with more than 30% sequence identity were used for comparison, and sequences with more than 60% sequence similarity were labeled with different colored backgrounds. These protein sequences were compared by the ClustalW program. The best model was calculated by selecting the NJ method via MUSCLE (Multiple Sequence Comparison by Log-Expectation). Then, the maximum likelihood phylogenetic tree was calculated by MEGA X. The phylogenetic test was calculated using bootstrap method. The number of replicates was calculated by bootstrap method with 1000. Finally, the tree was rendered by the iTOL online tool.

Interestingly, the structure of TglH resembles that of many TIM barrel-related enzymes,^43–51^ implying a common ancestral heritage. Therefore, evolutionary analysis of these enzymes was carried out using the maximum likelihood method (Figure 6A). The results showed that the DUF692 family forms a distinct clade from other enzymes that attain the TIM-barrel fold. However, structural analysis revealed the presence of three extended loops above the center of L-rhamnose isomerase (PDB accession No. 1DE6),^43^ which assemble to form a closed anionic cavity to house metal ions (Mn and Zn) and the substrate (Figure S7). Such flexible loops have also been observed in the structures of other enzymes, including xylose isomerase (PDB accession No. 9XIA),^51^ mannonate dehydratase (PDB accession No. 3FVM),^47^ and D-psicose 3-epimerase (PDB accession No. 2HK1).^50^ Therefore, despite low sequence identity compared to TglH, these proteins may undergo similar conformational changes during enzymatic activation and suggests that TglH retained structural features from other TIM barrel-related enzymes through evolution. Further bioinformatic analysis of representative enzymes from different organisms with over 30% sequence identity to TglH showed that nearly half of the homologs are derived from high G+C content gram-positive bacteria, with most of these having relatively low sequence identity. Interestingly, homologs with high sequence identity to TglH are widely distributed across alpha, beta, and gamma-proteobacteria and enterobacteria (Figure 6B).

In summary, our study provides new insights into the molecular mechanism of the formation of TglA-Cys^ΔCH2^ during biosynthesis of 3-thiaGlu and illuminates the similarities of and differences between the holoenzymes MbnBC and TglHI.

## STAR★Methods

### Resource availability

#### Lead Contact

Further information and requests for resources should be directed to and will be fulfilled by the lead contact, Wei Cheng (chengwei669@scu.edu.cn).

#### Materials Availability

This study did not generate new unique reagents.

#### Data and Code Availability

The coordinates and structure factors of apoTglHI, TglHI-2Fe and TglHI-3Fe have been deposited at the RCSB Protein Data Bank with accession No. 8HCI, 8HI7 and 8HI8, respectively.

This paper does not report original code.

Any additional information required to reanalyze the data reported in this paper is available from the lead contact upon request.

### Experimental model and subject details

#### *E. coli* competent cells

The commercially available *E. coli* DH5α and *E. coli* BL21(DE3) competent cells were used in the study. Unless otherwise stated these were grown in LB supplemented with suitable antibiotics, at 37°C and shaken at 220 rpm.

### Method details

#### Protein expression and purification

The *tglH* (NCBI accession No. WP_007252215.1) and *tglI* (NCBI accession No. WP_007252216.1) genes from *Pseudomonas syringae pv. maculicola* (*P. syringae*) ES4326 were codon-optimized for expression in *E. coli* (the optimized sequences are provided in KRT) and subcloned into the pRSFDuet-1 vector. *E. coli* BL21 (DE3) cells containing the *tglHI*-pRSFDuet-1 plasmid were grown in Luria broth (LB) medium at 37 °C and 220 rpm to an optical density at 600 nm (OD_600_) of 0.6. Fe-containing TglHI proteins were obtained by the addition of 1 mM (NH_4_)_2_Fe(SO_4_)_2_ in LB medium. Subsequently, cultures were induced at 16 °C with the addition of isopropyl *β*-D-thiogalactoside (IPTG) at a final concentration of 0.2 mM. Cell pellets were collected by centrifugation at 4 °C and 4000 rpm for 12 min and resuspended in lysis buffer (25 mM HEPES, pH 8.0, 300 mM NaCl). After cell lysis, lysates were centrifuged at 18000 rpm for 30 min to obtain the supernatant containing the target protein. Next, the supernatant was passed through a nickel affinity column (Ni-NTA; GE Healthcare, Little Chalfont, UK), the resin was cleaned with lysis buffer mixed with different concentrations of imidazole (10, 20 and 30 mM), and finally, the protein of interest was eluted with lysis buffer supplemented with 300 mM imidazole. The eluted protein was further purified by anion exchange columns (HiTrap Q HP; Cytiva) and size exclusion chromatography columns (Superdex200 Increase 10/30 GL; GE Healthcare, Sweden) and finally preserved in buffer A (15 mM HEPES, pH 8.0 and 150 mM NaCl). The purity of the purified proteins was assessed using sodium dodecyl sulfate‒polyacrylamide gel electrophoresis (SDS‒PAGE) and they were flash-frozen with liquid nitrogen for subsequent crystallization and biochemical analysis. In this study, pH 8.0 buffer was mainly used for crystallization, while pH 7.5 buffer was used for activity assays.

#### Preparation of iron-free TglHI

The *tglHI-pRSFDuet-1* plasmids were introduced into *E. coli* BL21 cells through a transformation process. Several clones were selected and cultured overnight in 100 mL of LB medium. Subsequently, 50 mL of the bacterial culture was transferred to a modified medium based on the minimal nutrient M9 medium (the recipe for M9 can be found in Table S3) and incubated for 2 h. The cells were then harvested by centrifugation at 4 °C and 4000 rpm for 12 minutes, followed by resuspension in a lysis buffer. The resuspended cells were then transferred to M9 expression medium. Once the OD_600_ reached 0.6, a final concentration of 4.5 mM sodium hydroxide was added. After cooling the mixture to 22 °C, amino acids were added and allowed to incubate for 30 minutes. The temperature was then further reduced to 16 °C, and IPTG was added at a final concentration of 0.15 mM to induce protein expression for 16 hours. Finally, the proteins were purified as described above.

#### TglHI activity measurement

To assess the activity of TglHI, a coupled assay with formate dehydrogenase (FDH) from *Candida boidinii* was optimized.^28^ The TglHI enzyme (25 μM) was incubated with the minimum substrate analog of TglA-Cys (100 μM), *β*-NAD^+^ (200 μM), and FDH (25 μM) in reaction buffer (15 mM HEPES, 100 mM NaCl, pH 7.5). The reaction was carried out at 25 °C, the course of the reaction was monitored by UV‒vis spectroscopy, and the absorbance of NADH production was continuously monitored at 340 nm.

#### Isothermal titration calorimetry (ITC)

The purified protein was thawed and diluted to a final concentration of 0.1 mM for ITC determination. The synthesized TglA-Cys analog containing the C-terminal 19 amino acids was dissolved in buffer A and diluted to a final concentration of 1.4 mM. The affinity of TglHI and TglA-Cys analog was determined by titration of the peptide into TglHI using a Nano ITC (Nano ITC- low volume; TA Instruments, USA). The optimal settings of the peptide-TglHI binding assay were as follows: the titration was carried out at 16 °C, and 2.5 μL of TglA-Cys peptide analog was injected into the sample pool containing 300 μL of TglHI protein (0.1 mM) every 120 s for 20 injections in total. The mixing speed of the blender syringe was set at 250 rpm. The raw titration dataset was analyzed using in single-point binding mode and then processed by NanoAnalyze Data Analysis software version 3.8.0.

#### Pull-down assay for the TglHIA-Cys complex

Full-length TglA-Cys was subcloned into the PGEX-6P-1 vector with a glutathione-*S*-transferase (GST) tag at the N-terminus of TglA-Cys and purified by a GST affinity column. The pull-down assay of TglHI and TglA-Cys was performed by incubating purified TglHI with GST-TglA-Cys for two hours in a GST column. Then the column was washed to remove unbound protein. The resins containing TglA-Cys before and after the addition of purified TglHI were resuspended and analyzed by SDS-PAGE.

#### Crystallization and data collection

Purified proteins (approximately 20 mg/mL) with and without iron were used for crystal growth. Protein crystals were preliminarily screened in Index (Hampton Research, USA), PEG/ION (Hampton Research, USA), Salt (Hampton Research, USA), Crystal (Hampton Research, USA), WIZard1/2 (Hampton Research, USA) and WIZard3/4 (Hampton Research, USA) kits by the sitting-drop vapor-diffusion method at 16 °C. Each well was filled with a mixture containing 1 μL of protein solution and the same amount of reservoir solution.

Crystals were obtained under a variety of conditions. The crystal of the resolved structure was obtained in a solution containing 0.2 M potassium sodium tartrate tetrahydrate and 20% w/v polyethylene glycol 3350. The crystal grew to a suitable size for X-ray diffraction after a week. Before X-ray diffraction, the crystals were immediately frozen in liquid nitrogen with a cryoprotectant containing 15% glycerol. X-ray diffraction data were collected by a Pilatus36 M detector at the Synchrotron Radiation Facility 18U1 in Shanghai, China. With an exposure time of 0.2 s and a distance of 400 mm from the crystal to the detector, a total of 360 images were captured using 1.0 oscillations. Collected data were indexed, integrated, and scaled using the HKL3000 software suite^52^ and CCP4i2.^53^ The crystal structure of TglHI was solved by molecular replacement via a model predicted by AlphaFold2.^37^ Next, the optimal solution was manually built in Coot and refined in PHENIX.^54–56^ The final models were validated by MolProbity and deposited in the Protein Data Bank (see Table 1).

#### Preparation of TglHI-2Fe and TglHI-3Fe

To produce TglHI-2Fe, the *tglHI-pRSFDuet-1* plasmid was introduced into *E. coli* BL21 cells, which were then cultivated in LB medium. In the case of TglHI-3Fe, both the *tglHI-pRSFDuet-1* and *tglA-Cys-PGEX-6P-1* plasmids without the GST tag were co-transformed into *E. coli* BL21 cells, followed by growth in LB medium. Prior to incubation, the LB medium was supplemented with 1 mM ferrous ammonium sulfate. All proteins were next expressed and purified as described above.

#### Fe content determination

ICP‒OES measurements were performed to determine how much Fe was in the complexes. For predigestion, 8 mL of nitric acid was combined with the TglHI and TglHIA-Cys proteins for 15 min. The ICP‒OES apparatus was then loaded with the supernatant for the determination of the Fe, Co, Mn and Cu contents.

#### TglHI activity assay followed by H_2_O_2_ or EDTA treatment

TglHI protein and H_2_O_2_ (or EDTA) were initially incubated together for 10 min, and then protein was purified by size exclusion chromatography to remove excess H_2_O_2_ (or EDTA). The reaction between the H_2_O_2_ (EDTA)-treated TglHI and minimum substrate analog of TglA-Cys, *β*-NAD^+^, and FDH was then monitored for the levels of NADH.

#### Site-directed mutagenesis

For each TglH and TglI mutant, the primer pairs listed in the Table S4 was used to circularized *tglHI-pRSFDuet-1* with *Taq* 2 x Master Mix (NEB). The PCR product was used to transform *E. coli* DH5α. Plasmids of TglH and TglI mutants were purified by TIANprep Mini Plasmid Kit and were validated using Tsingke sequencing. The preparation and purification of all the mutants were carried out using the same procedure as that for the native proteins.

#### Mass spectrometry analysis of His_6_-TglA-Cys co-expressed with TglH mutants and TglI

pACYCDuet-His_6_-*tglA*-*Cys* and pET15b-His_6_-*tglH*-*tglI* with inserts encoding alanine mutations of the Fe ligands were used to co-transform *E. coli* BL21 cells using the KCM method. Cells were selected on LB agar medium supplied with 100 mg/L carbenicillin and 25 mg/L chloramphenicol and grown at 37 °C until colonies appeared. A single colony was used to inoculate 5 mL of LB medium supplied with the same concentration of carbenicillin and chloramphenicol. The starter culture was grown at 37 °C for 12–16 h. Then 1 mL of the starter culture was used to inoculate 100 mL of LB medium supplied with the same concentration of carbenicillin and chloramphenicol. The 100 mL culture was incubated at 37 °C 220 rpm until OD_600_ reached 0.6–1. The culture was cooled at 4 °C for 30 min before the protein overexpression was induced by adding 0.2 mM (final concentration) IPTG. The culture was incubated at 18 °C and shaken at 220 rpm for another 20 h.

*E. coli* BL21 cells were harvested by centrifugation at 4000x*g* for 5 min at 10 °C and were resuspended in 10 mL of purification buffer (50 mM HEPES, 300 mM NaCl, 10% glycerol, pH 7.5). The *E. coli* BL21 cells were lysed by sonication with 1 s on, 5 s off cycles at 15% amplitude for a total of 5 min. The lysate was clarified by centrifugation at 12500x*g* for 20 min. The supernatant was supplied with 0.5 mM tris (2-carboxyethyl) phosphine (TCEP), 10 mM imidazole, and Ni-resin (Takara). The mixture was nutated at 4 °C for 10 min to allow binding of proteins with His_6_-tag. The resin was harvested by centrifugation at 2000x*g* for 3 min. The resin was resuspended in the purification buffer supplied with 30 mM imidazole and was loaded onto a gravity column. The resin was washed with 20 mL of 30 mM imidazole in the purification buffer and 10 mL of 50 mM imidazole in the buffer. The bound peptide was then eluted using 2 mL of the purification buffer supplied with 500 mM imidazole.

Ziptip C18 (Sigma) was used to desalt the eluted peptide. The bound peptide was eluted using 10 μL of 60% acetonitrile + 0.1% trifluoroacetic acid. Then 1 μL of the desalted peptide solution was spotted with 1 μL 50 mg/mL super-DHB (Sigma) onto a mass spectrometry target plate. Matrix-assisted laser absorption/desorption ionization-time-of-flight mass spectrometry (MALDI-TOF-MS) analysis was performed using a Bruker UltrafleXtreme MALDI TOF-TOF mass spectrometer (Bruker Daltonics) at the University of Illinois School of Chemical Sciences Mass Spectrometry Laboratory.

For the N187A, H216A, and D229A mutants of TglH, the products appeared to have a wider isotope distribution. Thus, these peptides were digested and analyzed by high resolution electron spray ionization MS (HRMS). For this assay, 1 mL of the imidazole-eluted peptide from Ni-NTA purification was reacted with 1 mM TCEP and 10 mM iodoacetamide at room temperature for 1 h to alkylate the thiol in the TglA-Cys^ΔCH2^. The alkylated peptide solution was exchanged into 50 mM ammonium bicarbonate three times using 3 kDa molecular weight cutoff filters (Sigma). Three micrograms of sequencing grade trypsin (Worthington) were added to 100 μL of peptide solution. The trypsin digest was allowed to proceed at 37 °C for 3 h. The digest was purified using a TopTip C-18 column that had been wetted with 60% acetonitrile + 0.1% formic acid and equilibrated with 0.1% formic acid in H_2_O. Tryptic fragments were eluted with 60% acetonitrile + 0.1% formic acid, and this solution was directly used for HPLC-HRMS and MS/MS experiments. The regular TglHI product was observed (Figure S8D and S8E) and no other products were observed. We do not know why the MALDI-TOF MS analysis appears to show additional products. These may have been contaminants from *E. coli* BL21. The column used for separation was an Agilent C18 AdvanceBio Peptide Plus 2.1 × 150 mm, 2.7 μm, and was maintained at 45 °C for all analysis.

MS parameters were as follows. Ion polarity: positive; mass range: 50–1700 m/z; slicer mode: high resolution; gas temperature: 325 °C; drying gas: 10 L/min; nebulizer pressure: 35 psi; sheath gas temperature 375 °C; sheath gas flow 11 L/min; capillary voltage: 3500 V; nozzle voltage: 0 V; fragmentor voltage: 120 V; skimmer: 65 V; Oct 1 RF Vpp: 750 V. Acquisition mode: targeted-MS/MS. Acquisition rate: MS^1^: 10 Hz, MS^2^: 5 Hz. Isolation width: 1.3 m/z. Collison energy: 10 eV.

HPLC parameters were as follows: flow rate 0.4 mL/min. Mobile Phase A was H_2_O + 0.1% formic acid and mobile phase B was acetonitrile + 0.1% formic acid. The solvent gradient was: 0–2 min: 2% B; 7 min: 20% B; 12 min: 60% B; 13 min: 100% B; 13.5 min: 2% B. Re-equilibration was achieved with 3 min at 2% B between injections.

Data analysis was performed using Agilent MassHunter Qualitative Analysis 10.0. The m/z lists for MS^2^ spectra were exported for figure generation using Interactive Peptide Spectral Annotator.

#### Sequence alignment and homology clustering

The ClustalW program was used to create the multiple sequence alignments, and the ESPript 3.0^57^ was used to modify them. The TglH homologs and TIM-related enzymes were found by BLAST with the NCBI and EMBL-EBI databases, respectively. The evolutionary tree was constructed by selecting the NJ method through the multiple sequence comparison by log-expectation (MUSCLE) multiple sequence alignment algorithm in MEGA X. The selected test method was the bootstrap method (self-extension test method) with 1000 tests, and the Poisson protein sequence substitution model was selected to finally construct the phylogenetic tree of TglH. Finally, MEGA X,^58^ with iTOL editing,^59^ constructed the greatest likelihood phylogenetic tree.

### Quantification and statistical analysis

The number for each replica and details of statistical analyses are described in the figure legends.

## Supplementary Material

supplemental information

## Figures and Tables

**Table 1.** Data collection and refinement statistics

	apoTglHI	TglHI-2Fe	TglHI-3Fe
	(PDB ID: 8HCI)	(PDB ID: 8HI7)	(PDB ID: 8HI8)
**Data collection**			
Resolution range (Å)	61.15–3.39	101.9–3.25	29.74–3.49
	(3.52–3.39)^a^	(3.37–3.254)^a^	(3.61–3.49)^a^
Unit cell	77.9522	117.63	118.943
	86.3136	117.63	118.943
	91.9944	86.7631	87.46
	90	90	90
	109.66	90	90
	90	120	120
Space group	P 1 2_1_ 1	P 6_1_	P 6_1_
Completeness (%)	98.32 (99.43)^a^	99.34 (100.00)^a^	99.49 (99.89)^a^
Multiplicity	3.3 (3.5)^a^	11.2 (11.3)^a^	19.2 (19.5)^a^
No. of unique reflections	15758 (1580)^a^	10784 (1068)^a^	9013 (899)^a^
Mean I/sigma (I)	5.58 (3.47)^a^	11.98 (3.70)^a^	18.95 (5.80)^a^
CC_1/2_	0.958 (0.861)^a^	0.995 (0.832)^a^	0.999 (0.958)^a^
R_merge_	0.1568 (0.331)^a^	0.1808 (0.771)^a^	0.1559 (0.7585)^a^
**Refinement**			
R_free_/R_work_	0.25/0.22	0.24/0.20	0.25/0.20
No. of non-hydrogen atoms	9163	4597	4617
Proteins	9116	4556	4556
Ligands	0	2	3
Solvent	47	39	58
Ramachandran allowed (%)	3.12	5.54	6.61
Ramachandran favored (%)	96.70	94.46	93.39
Ramachandran outliers (%)	0.18	0.00	0.00
Rotamer outliers (%)	0.00	0.00	0.00
RMS (bonds)	0.003	0.002	0.002
RMS (angles)	0.60	0.50	0.48
Average B-factor	75.54	74.37	109.85
Proteins B-factor	75.52	74.47	109.95
Ligands B-factor	N/A	77.89	123.70
Solvent B-factor	79.40	61.89	101.58

a Statistics for the highest-resolution shell are shown in parentheses.

## Inclusion and Diversity

We support inclusive, diverse, and equitable conduct of research.
