# Supplementary material for: Structures of the holoenzyme TglHI required for 3-thiaglutamate biosynthesis
Source: Structure. Author manuscript; Available in PMC 2024 Feb 21. (PMC10880893; doi:10.1016/j.str.2023.08.004)
Supplement: supplemental information [file NIHMS1963473-supplement-supplemental_information.pdf]

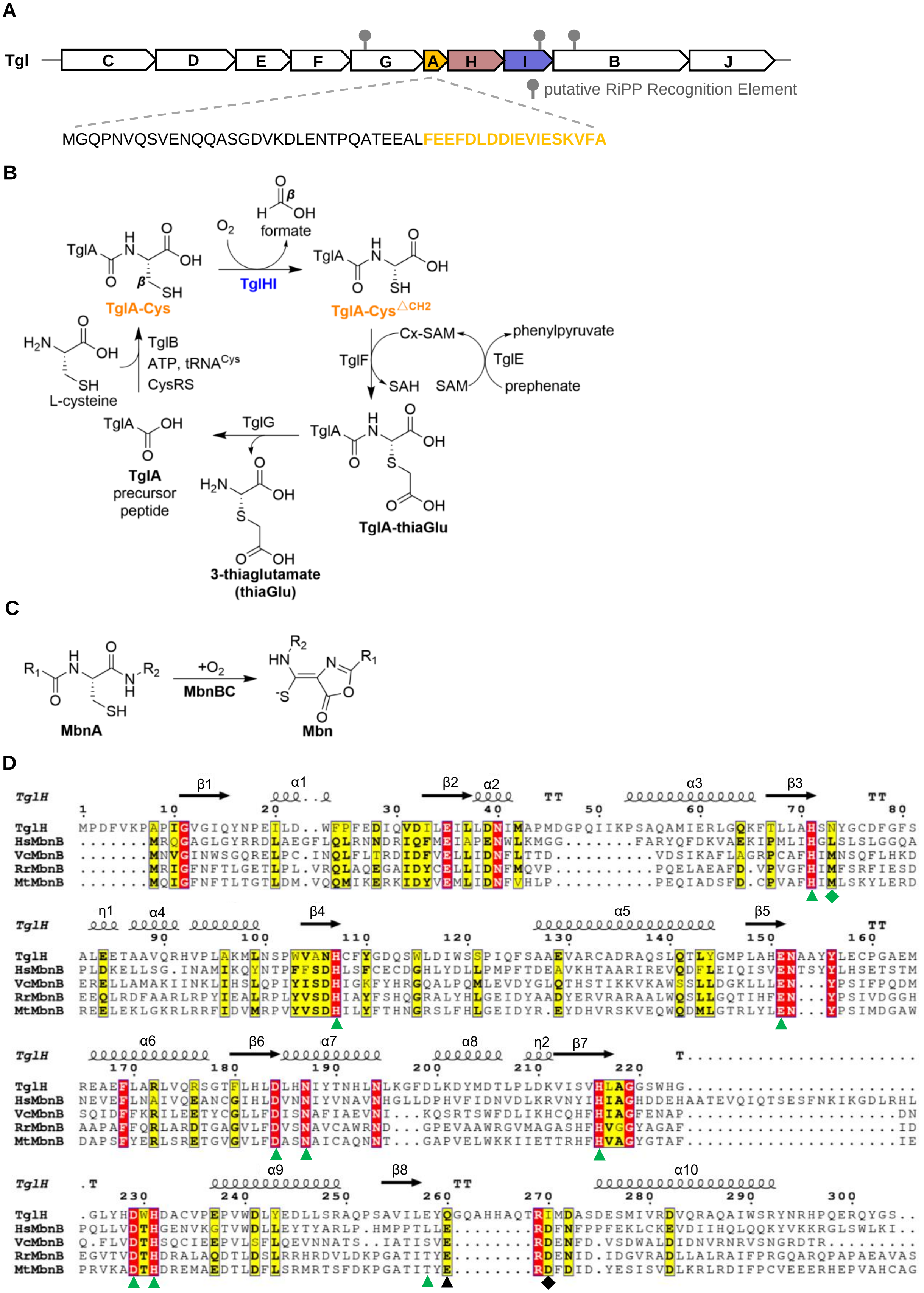

A
Tgl
C D E F G A H I B J
putative RiPP Recognition Element
MGQPNVQSVENQQASGDVKDLENTPQATEEALFEEFDLDDIEVIESKVFA
B
TglA-Cys
O2
formate
TglHI
TglA-Cys△CH2
Cx-SAM
phenylpyruvate
TglF
TglE
SAH SAM
prephenate
TglB
ATP, tRNACys
CysRS
L-cysteine
TglG
TglA
precursor
peptide
TglA-thiaGlu
3-thiaglutamate
(thiaGlu)
C
+O2
MbnBC
MbnA
Mbn
D
TglH
MPDFVKPAPIGVGIQYNPEILD..WFPFEDIQVDILEILLDNIMAPMDGPQIIKPSAQAMIERLGQKFTLLAHSNYGCDFGFS
HsMbnB .......MRQGAGLGYRRDLAEGFLQLRNNDRIQFMEIAPENWLKMGG.......FARYQFDKVAEKIPMLIHGLSLSLGGQA
VcMbnB .......MNVGINWSGQRELPC.INQLFLTRDIDFVELLIDNFLTTD.........VDSIKAFLAGRPCAFHIMNSQFLHKD
RrMbnB .......MRIGFNFTLGETLPL.VRQLAQEGAIDYCELLIDNFMQVP..........PQELAEAFD.VPVGFHIMFSRFIESD
MtMbnB .......MQIGFNFTLTGTLDM.VQQMIKERKIDYVEMLIDNFVHLP..........PEQIADSFD.CPVAFHIMLSKYLERD
TglH ALEETAAVQRHVPLAKMLNSPWVANHCFYGDQSWLDIWSSPIQFSAAEVARCADRAQSLQTLYGMPLAHENAAYYLECPGAEM
HsMbnB PLDKELLSG.INAMIKQYNTPFFSDHLSFCECDGHLYDLLPMPFTDEAVKHTAARIREVQDFLEIQISVENTSYYLHSETSTM
VcMbnB ERELLAMAKIINKLIHSLQPIYISDHIGKFYHRGQALPQMLEVDYGLQTHSTIKKVKAWSSLLDGKLLLEN...YPSIFPQDM
RrMbnB EEQLRDFAARLRPYIEALRPLYVSDHIAYFSHQGRALYHLGEIDYAADYERVRARAALWQSLLGQTIHFEN...YPSIVDGGH
MtMbnB REELEKLGKRLRRFIDVMRPVYVSDHILYFTHNGRSLFHLGEIDYR.EYDHVRSKVEQWQDMLGTRLYLEN...YPSIMDGAW
TglH REAEFLARLVQRSGTFLHLDLHNIYTNHLNLKGFDLKDYMDTLPLDKVISVHLAGGSWHG.......................
HsMbnB NEVEFLNAIVQEANCGIHLDVNNIYVNAVNHGLLDPHVFIDNVDLKRVNYIHIAGHDDEHAATEVQIQTSESFNKIKGDLRHL
VcMbnB SQIDFFKRILEETYCGLLFDISNAFIAEVNI...KQSRTSWFDLIKHCQHFHIAGFENAP.....................DN
RrMbnB AAPAFFQRLARDTGAGVLFDVSNAVCAWRND...GPEVAAWRGVMAGASHFHVGGYAGAF.....................ID
MtMbnB DAPSFYERLSRETGVGVLFDASNAICAQNNT...GAPVELWKKIIETTRHFHVAGYGTAF.....................IE
TglH .GLYHDWHDACVPEPVWDLYEDLLSRAQPSAVILEYQGQAHHAQTRIMDASDESMIVRDVQRAQAIWSRYNRHPQERQYGS..
HsMbnB PQLLVDTHGENVKGTVWDLLEYTYARLP.HMPPTLLE........RDFNFPPFEKLCKEVDIIHQLQQKYVKKRGLSWLKI..
VcMbnB Q.FLVDTHSQCIEEPVLSFLQEVNNATS..IATISVE........RDENFD.VSDWALDIDNVRNRVSNGRDTR.........
RrMbnB EGVTVDTHDRALAQDTLDSLRRHRDVLDKPGATITYE........RDENID.IDGVRADLLALRAIFPRGQARQPAPAEAVAS
MtMbnB PRVKADTHDREMAEDTLDFLSRMRTSFDKPGATITYE........RDFDID.YESISVDLKRLRDIFPCVEEERHEPVAHCAG

Figure S1. 3-Thiaglutamate (3-thiaGlu) biosynthesis in *Pseudomonas syringae pv. maculicola str. ES4326* , and sequence alignment of TglH and MbnB required for methanobactin (Mbn) biosynthesis (Related to Figure 1)

(A) The *tglH* (pink) and *tglI* (slate) genes are colored in the *tgl* biosynthetic gene cluster; sites encoding RiPP recognition element (RRE) domains are indicated in gray. The minimum substrate analog of TglA is highlighted in orange.

(B) Role of TglHI in the biosynthesis of 3-thiaGlu. First, TglB forms TglA-Cys by cysteinylating the ribosomally translated precursor peptide TglA (orange) at its phosphorylated C-terminus in an ATP- and Cys-tRNA^{Cys}-dependent manner. After that, the oxygen-dependent excision of the Cys methylene group is catalyzed by the TglHI complex, releasing the Cβ as formate and generating an intermediate containing an alpha-thiol group. The C-terminal secondary thiol is then modified by a carboxy-SAM (Cx-SAM)-dependent methyltransferase called TglF to yield TglA-thiaGlu. The C-terminal 3-thiaglutamate is then released by the membrane-associated protease TglG, releasing TglA as a scaffold for the following biosynthesis cycle.

(C) Biosynthesis of Mbn from MbnA catalyzed by MbnBC. The oxazolone-thioamide installation is executed by MbnBC-catalyzed MbnA modification, in which the MbnA^{Cys} sulfhydryl group is ligated to one of the irons of the tri-iron center of dioxygen-dependent MbnBC holoenzyme.

(D) Amino acid sequence alignment of TglH, HsMbnB (*Histophilus somni,* WP_249962398.1), VcMbnB (*Vibrio caribbenthicus*, WP_009601368.1), RrMbnB (*Rugamonas rubra*, WP_093383377.1) and MtMbnB (*Methylosinus trichosporium*, WP_065083569.1). Residues in TglH that involve coordination to Fe are indicated by green triangles (Structural superposition demonstrates that E258 in TglH corresponds to E238 in VcMbnB or E240 in RrMbnB with a black triangle), and a key residue for catalysis is indicated by a green diamond. The Asp that has been proposed to serve as a general base in MbnB is indicated in a black diamond. Fully conserved residues are highlighted in red and mostly conserved residues are highlighted in yellow. For many of the yellow highlighted residues, the amino acid in TglH is different from that in MbnB.

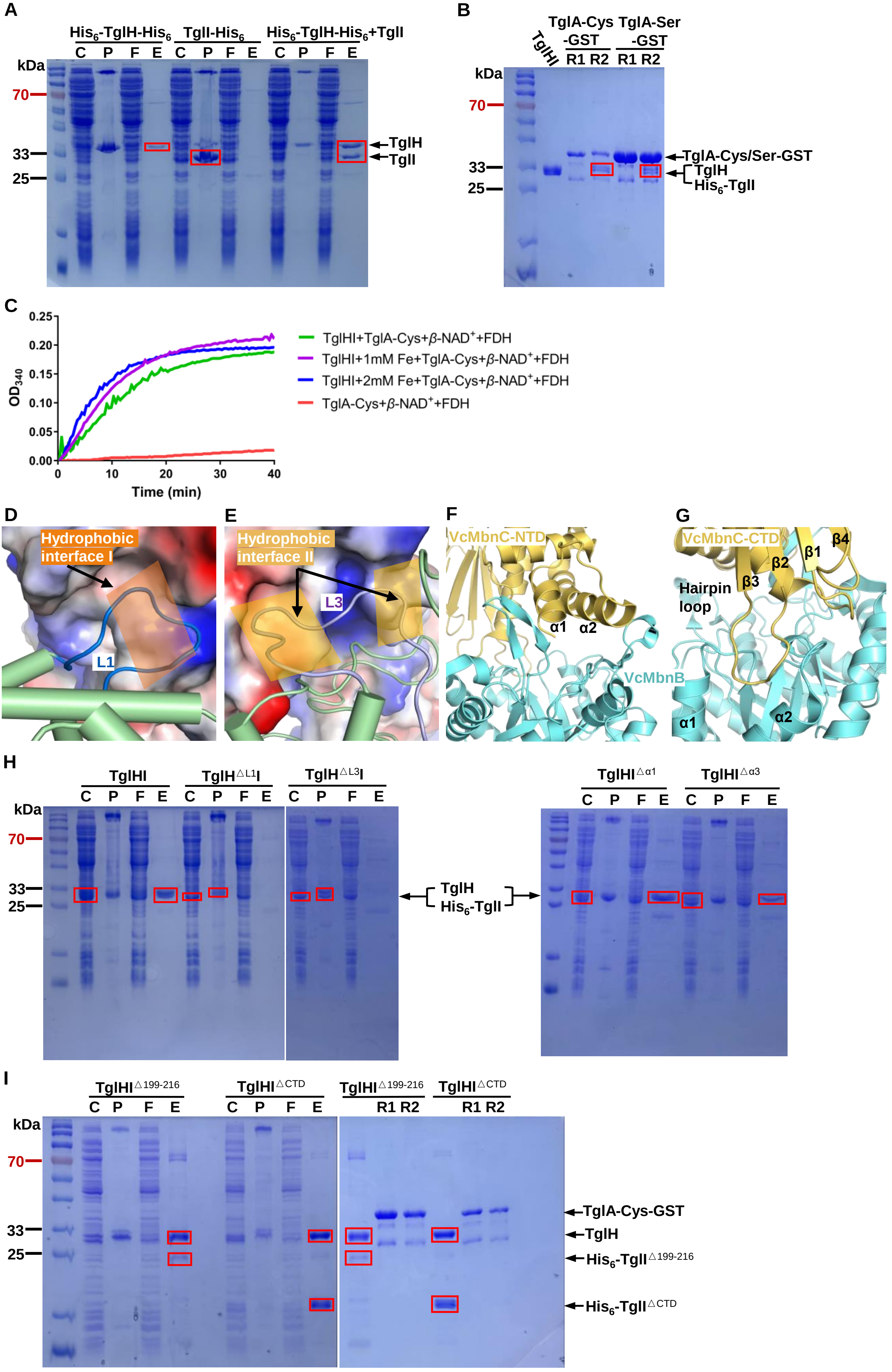
A
His6-TglH-His6
TglI-His6
His6-TglH-His6+TglI
C P F E C P F E C P F E
kDa
70
33
25
TglH
TglI
B
TglHI
TglA-Cys -GST
TglA-Ser -GST
R1 R2 R1 R2
kDa
70
33
25
TglA-Cys/Ser-GST
TglH
His6-TglI
C
OD340
0.25
0.20
0.15
0.10
0.05
0.00
0 10 20 30 40
Time (min)
TglHI+TglA-Cys+β-NAD+ +FDH
TglHI+1mM Fe+TglA-Cys+β-NAD+ +FDH
TglHI+2mM Fe+TglA-Cys+β-NAD+ +FDH
TglA-Cys+β-NAD+ +FDH
D
Hydrophobic interface I
L1
E
Hydrophobic interface II
L3
F
VcMbnC-NTD
α1 α2
VcMbnB
G
VcMbnC-CTD
β1 β2 β3 β4
Hairpin loop
α1 α2
H
TglHI
TglH△L1I
TglH△L3I
TglHI△α1
TglHI△α3
C P F E
kDa
70
33
25
TglH
His6-TglI
I
TglHI△199-216
TglHI△CTD
C P F E
R1 R2
kDa
70
33
25
TglA-Cys-GST
TglH
His6-TglI△199-216
His6-TglI△CTD

Figure S2. Pull-down assay for the TglHI and TglA-Cys complexes, Interaction between apoTglH and TglI (Related to Figure 2)

(A) SDS-PAGE of TglH and TglI when expressed separately. *tglH* and *tglI* genes were inserted into the pET28b vector and pET21b vector, respectively. *tglH*-pET28b construct contains both N-terminal and C-terminal His_6-tags, whereas the *tglI*-pET21b construct contains only the C-terminal His_6-tag. When TglH and TglI were co-expressed, the His_6 tag at the C-terminal end of TglI was removed.

(B) The *tglH* and *tglI* genes were inserted into the pRSF-DuET-1 vector for co-expression, and the complexes were purified through nickel affinity columns. The full-length TglA-Cys protein carrying the GST tag was purified and was bound to a GST affinity resin. Subsequently, TglA-Cys-bound resin was incubated with the purified TglHI protein for 2 h and then the column was washed. The proteins were then eluted and analyzed by SDS-PAGE.

(C) *In vitro* activity assay of the modification of the minimum substrate analog of TglA-Cys by TglHI expressed in *E. coli* BL21 grown in LB medium supplemented without (green) and with (purple, blue) different concentration of ammonium ferrous sulfate in the presence of formate dehydrogenase (FDH) and β-NAD^+.

(D) Hydrophobic interface I formed by L1 (residues 41-53) of apoTglH with TglI. The electrostatic potential in all figures was computed using the APBS tools in PyMol (http://www.pymol.org/).

(E) Hydrophobic interface II formed by L3 (residues 151-166) of apoTglH with TglI.

(F) Interaction between VcMbnB and the N-terminal domain (NTD) of VcMbnC (PDB accession No. 7DZ9). The NTD of MbnC is sandwiched between two extended loops of MbnB.

(G) Interaction between VcMbnB and the C-terminal domain (CTD) of VcMbnC (PDB accession No. 7DZ9). The hairpin loop between β2 and β3 of VcMbnC CTD contacts VcMbnB.

(H) Coomassie stained SDS-PAGE gel showing co-expressed TglHI, $TglH^{\Delta L1}I$, $TglH^{\Delta L3}I$, $TglHI^{\triangle \alpha 1}$ and $TglHI^{\triangle \alpha 3}$ complexes. The TglHI positions are indicated with red boxes. L3 in TglH engages in interactions with α1 and α3 in TglI.

(I) Coomassie stained SDS-PAGE gel showing co-expressed $TglHI^{\triangle 199\text{-}216}$ and $TglHI^{\triangle CTD}$, and pull-down assay of these mutant and TglA-Cys. The positions for TglHI mutants are indicated with red boxes. The destruction of the C-terminal domain (residues 152-269, CTD) of TglI in TglHI leads to the disappearance of the TglA-Cys interaction with TglHI. Abbreviations: C, crude; P, pellet; F, flow through; E, eluent; R1, resin before TglHI was added; R2, resin after TglHI addition and washing.

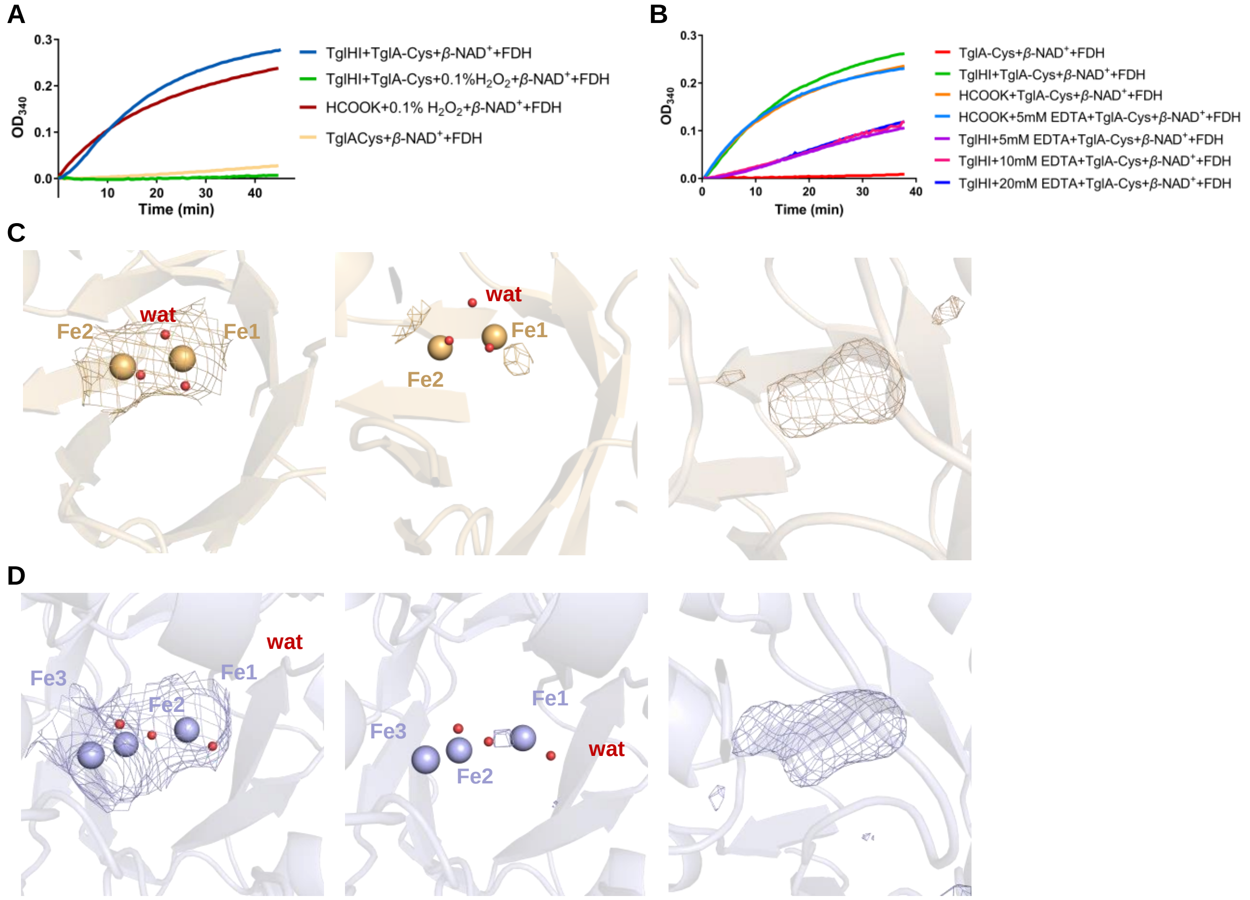

Figure S3. TglHI is an Fe-dependent holoenzyme (Related to Figure 3)

(A) *In vitro* activity assay of the modification of the minimum substrate analog of TglA-Cys by TglHI with treatment of H_2O_2 in the presence of formate dehydrogenase (FDH) and β-NAD^+. NADH formation was monitored at 340 nm. Activity was reduced to background levels by omission of TglHI (beige) or by addition of H_2O_2 (green). This latter observation was not the result of inactivation of FDH by H_2O_2 as shown by the maroon trace.

(B) *In vitro* activity assay of the modification of the minimum substrate analog of TglA-Cys by TglHI with treatment of EDTA (5 mM, 10 mM, 20 mM). Higher concentrations of EDTA will lead to precipitation of TglHI.

(C) Electron density maps contoured with a 2Fo-Fc map (left) at 1.0 σ and Fo-Fc map (middle) at 2.0 σ in the active center of TglH-2Fe. The right presents the Fo-Fc map contoured at 4.0 σ using the model without iron and waters in the active center of TglH-2Fe.

(D) Electron density maps with a 2Fo-Fc map at 1.0 σ and Fo-Fc map at 2.0 σ in the active center of TglH-3Fe. The right presents the Fo-Fc map contoured at 4.0 σ using the model without iron and waters in the active center of TglH-3Fe. Structure of TglHI-3Fe was obtained by co-expression with the substrate TglA-Cys and the addition of equivalent amounts of ammonium ferrous sulfate to the medium as stated for TglHI-2Fe. Fe irons are represented as spheres.

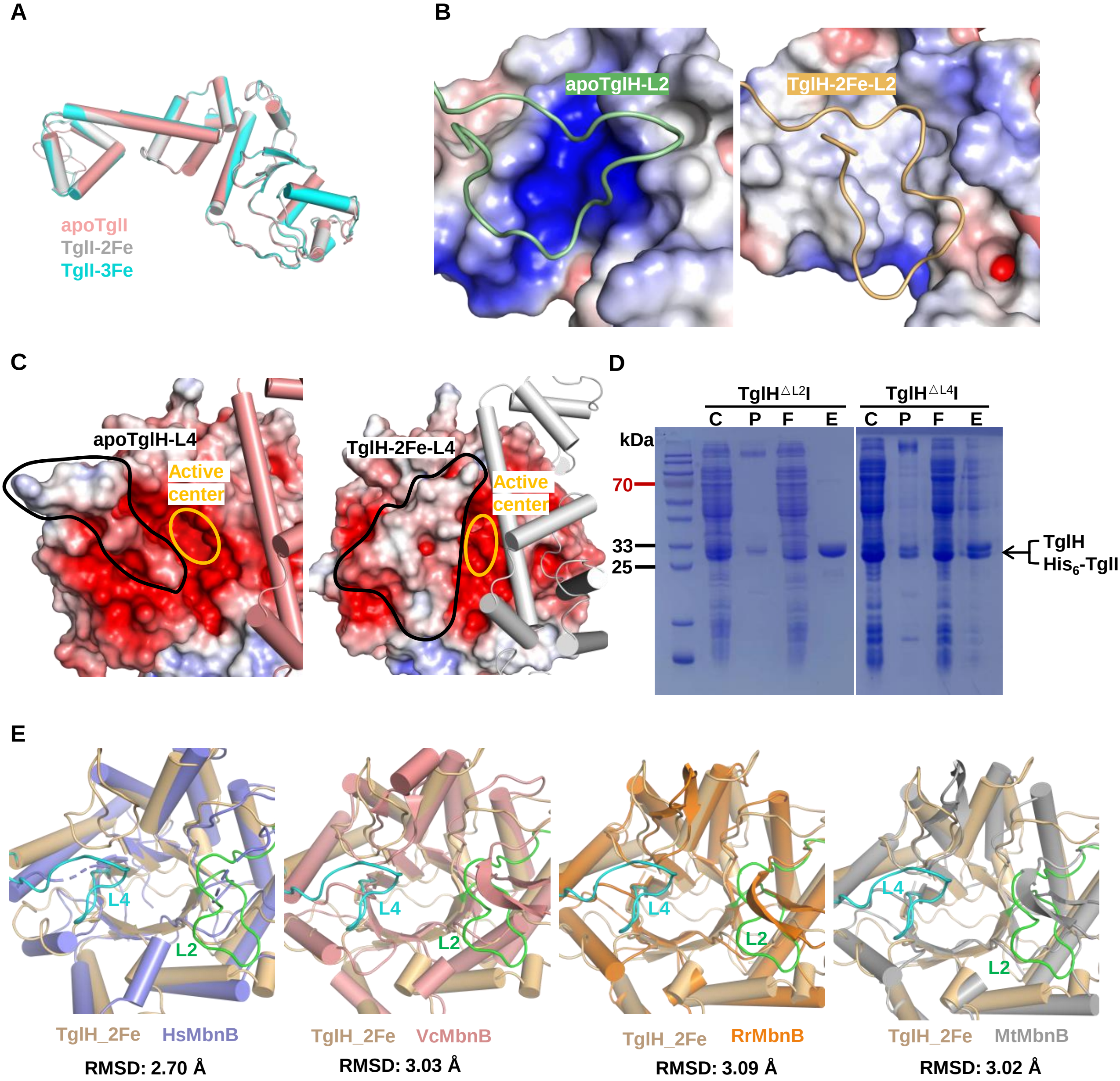

Figure S4. Structural comparison of apoTglHI and TglHI-2Fe (Related to Figure 3)

(A) Superposition of TglI structures from apoTglHI, TglHI-2Fe and TglHI-3Fe.

(B) Ineraction surfaces of apoTglH-L2 (left) and TglH-2Fe-L2 (right) with TglI, respectively. Fe irons binding causes TglH to undergo significant conformational changes resulting in an increase of hydrophobic interfaces. The electrostatic potential in all figures was computed using the APBS tools in PyMol (http://www.pymol.org/).

(C) Interaction surfaces of apoTglH-L4 (left) and TglH-2Fe-L4 (right) with TglI, respectively. The surface of the potential formed by L4 of TglH is highlighted with a solid black line.

(D) Coomassie staining SDS-PAGE gel showing co-expressed TglH$^{\Delta L2}$I and TglH$^{\Delta L4}$I complexes. Abbreviations: C, crude; P, pellet; F, flow through; E, eluent.

(E) Structural superposition of TglH and homologues HsMbnB (PDB accession No. 3BWW), VcMbnB of VcMbnABC (PDB accession No. 7DZ9), RrMbnB of RrMbnABC (PDB accession No. 7FC0) and (D) MtMbnB of MtMbnBC (PDB accession No. 7TCR). The flexible loops (L2/L4) involved in cavity formation of TglHI exist only in TglH. Compared to MbnB (except HsMbnB), L2 of TglH is replaced by a hairpin loop that is essential for MbnBC interaction. L2/L4 loops are disordered in HsMbnB.

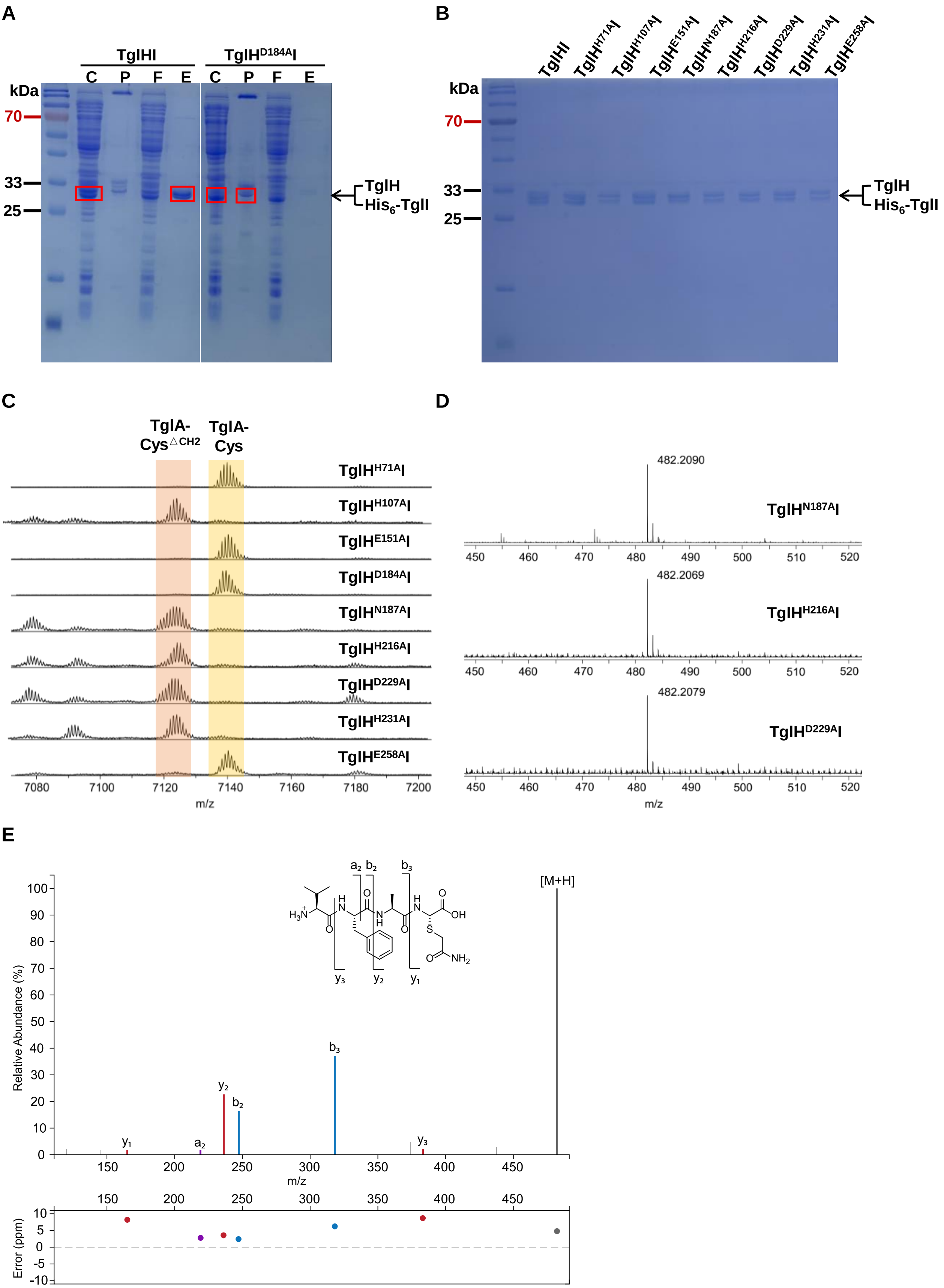

A
TglHI
TglH^{D184A}I
C P F E C P F E
kDa
70
33
25
TglH
His$_6$-TglI
B
TglHI
TglH^{H71A}I
TglH^{H107A}I
TglH^{E151A}I
TglH^{N187A}I
TglH^{H216A}I
TglH^{D229A}I
TglH^{H231A}I
TglH^{E258A}I
C
TglA-Cys$^{\triangle CH2}$
TglA-Cys
m/z
D
482.2090
482.2069
482.2079
m/z
E
[M+H]
Relative Abundance (%)
m/z
Error (ppm)

Figure S5. Mutagenesis of TglH active site residues (Related to Figure 4)
(A) Co-expression of TglH variant (D184A) with TglI as detected by SDS-PAGE.
(B) Co-expression of TglH active site mutants with TglI as detected by SDS-PAGE.
(C) TglH active site variants co-expressed with His_6-TglA-Cys and TglI followed by Ni-NTA purification and analysis of the peptide by MALDI-TOF MS. The N187A, H216A, and D229A products appear to contain another ion at slightly lower m/z as judged by the wider isotope envelope.
(D-E) Attempts to identify the N187A, H216A, and D229A products by S-alkylation with iodoacetamide followed by digestion with trypsin and analysis by HR ESI MS **(D)** showed only the regular product as demonstrated by tandem MS **(E)**. The identity of the additional mass observed in the MALDI-TOF mass spectrum is therefore unknown and could be derived from a peptide or protein from *E. coli*. Nevertheless, the data clearly show that these three TglH variants still produce the same product as wild type TglH.

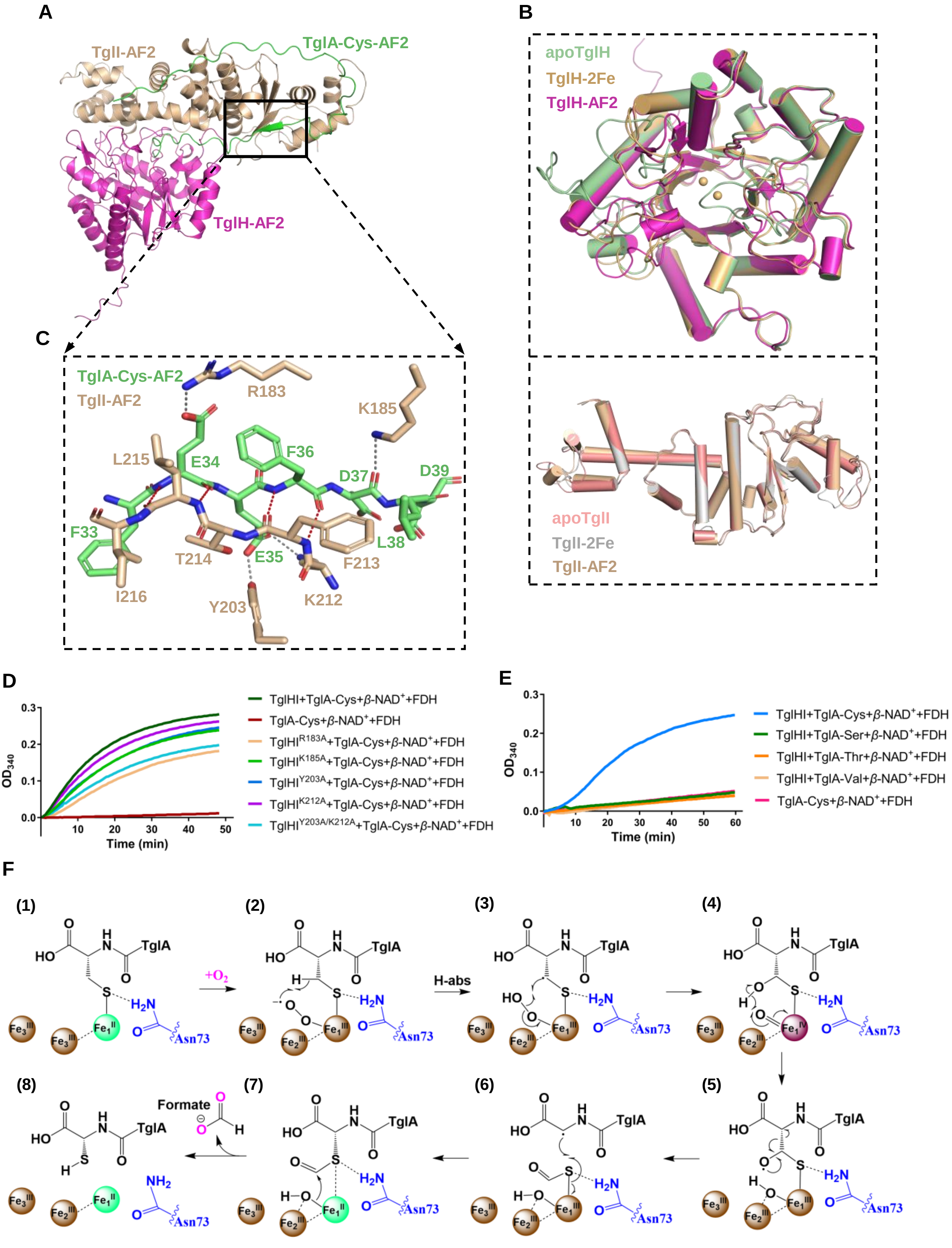

A
TglI-AF2
TglA-Cys-AF2
TglH-AF2
B
apoTglH
TglH-2Fe
TglH-AF2
apoTglI
TglI-2Fe
TglI-AF2
C
TglA-Cys-AF2
TglI-AF2
R183
K185
L215
E34
F36
D37
D39
F33
T214
E35
L38
F213
I216
Y203
K212
D
OD340
Time (min)
TglHI+TglA-Cys+β-NAD+ +FDH
TglA-Cys+β-NAD+ +FDH
TglHI R183A+TglA-Cys+β-NAD+ +FDH
TglHI K185A+TglA-Cys+β-NAD+ +FDH
TglHI Y203A+TglA-Cys+β-NAD+ +FDH
TglHI K212A+TglA-Cys+β-NAD+ +FDH
TglHI Y203A/K212A+TglA-Cys+β-NAD+ +FDH
E
OD340
Time (min)
TglHI+TglA-Cys+β-NAD+ +FDH
TglHI+TglA-Ser+β-NAD+ +FDH
TglHI+TglA-Thr+β-NAD+ +FDH
TglHI+TglA-Val+β-NAD+ +FDH
TglA-Cys+β-NAD+ +FDH
F
(1)
(2)
(3)
(4)
(5)
(6)
(7)
(8)
+O2
H-abs
TglA
Asn73
Formate

Figure S6. Specific recognition of TglA-Cys by TglHI (Related to Figure 5)
(A) Structure of TglHIA-Cys complex predicted by AlphaFold2 (named TglHIA-Cys-AF2).
(B) Superposition of TglH (top) and TglI (bottom) structures from apoTglHI, TglHI-2Fe and TglHI-AF2, respectively.
(C) The predicted mechanism of engagement of TglA-Cys with TglI. Four residues of TglA-Cys (Phe33, Glu34, Glu35, and Phe36) and TglI form a reverse β-strand through hydrogen bonds (shown in red dashes) between amide N-H and carbonyl oxygens on the backbone.
(D) *In vitro* activity of TglHI mutants involved in the interaction between the minimum substrate analog of TglA-Cys and TglI.
(E) *In vitro* activity of TglHI incubated with the minimum substrate analog of TglA-Cys, TglA-Ser, TglA-Thr and TglA-Val, respectively. The latter three peptides give responses that are indistinguishable from a negative control in which TglHI was omitted (pink) due to formate contamination, which is ubiquitous.
(F) Proposed mechanism of TglHI. The proposed carbon excision reaction catalyzed by TglHI begins with the activation of oxygen by Fe_1 that is also liganded by the C-terminal cysteine of TglA-Cys, but we cannot rule out that the oxygen interacts with one of the other irons, which would need to be in the ferrous form. Asn73 is required for activity and is likely important to orient Cys for coordination to Fe_1 by the formation of a hydrogen bond between the $-NH_2$ of its side chain amide and the thiolate of Cys. This orientation may also be important for hydrogen atom abstraction from the *β*-carbon of the Cys. As shown the mechanism implies that TglHI catalyzes dioxygenase activity but it has not been experimentally shown that both oxygens in formate are derived from O_2. The iron-bound hydroxide that hydrolyzes the formyl thioester in the final step could exchange with solvent leading to only one oxygen of O_2 ending up in formate.

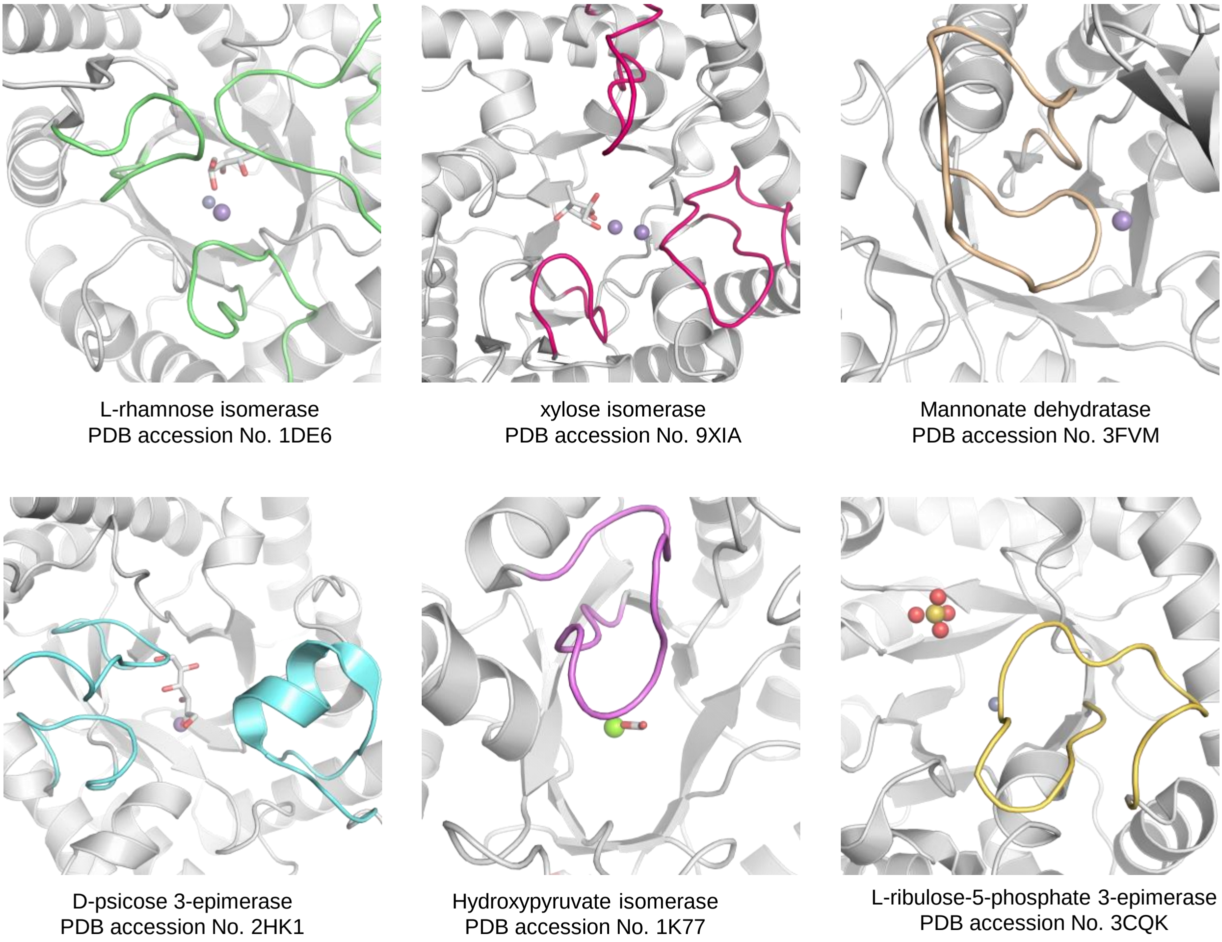

Figure S7. Structures of TIM-barrel-related enzymes (Related to Figure 6)
The active cavities of these enzymes are covered by flexible loops, a structural feature similar to that of TglH. This closed cavity, which is essential for enzyme activity, may reflect the evolutionary conservation of the structure.

Table S1. Fe content analysis for TglHI and TglHIA-Cys (Related to Figures 1 and 4)

Protein	Fe	Mn	Cu	Co
apoTglHI [a]	0.04±0.001	<0.01	<0.01	<0.01
TglHI	0.91±0.02	0.04±0.001	<0.01	<0.01
TglHIA-Cys	1.62±0.04	0.03±0.01	<0.01	<0.01
TglHI (1mM Fe)	1.50±0.09	0.02±0.001	<0.01	<0.01
TglHIA-Cys (1mM Fe)	2.48±0.18	0.03±0.01	<0.01	<0.01
TglHI (2mM Fe)	1.51±0.06	0.05±0.02	<0.01	<0.01
TglHIA-Cys (2mM Fe)	2.51±0.17	0.05±0.01	<0.01	<0.01

[a]The apoTglHI was expressed in in *E. coli* BL21 grown in M9 medium supplemented without ammonium ferrous sulfate. Other proteins were obtained in LB medium supplemented without and with different concentrations of ammonium ferrous sulfate (1mM, 2mM), respectively. All the values are mean and standard deviation of the measured metal concentrations for three independent protein preparations.

Table S2. Fe content analysis for TglH variants from TglHI or TglHIA-Cys complex (Related to Figures 3 and 4)

Protein	Fe	Mn	Cu	Co
TglH$^{\triangle 110\text{-}120}$I	0.78±0.01	<0.01	<0.01	<0.01
TglH$^{\triangle 263\text{-}271}$I	1.11±0.02	<0.01	<0.01	<0.01
TglH^{D229A}IA-Cys	1.32±0.02	0.02±0.01	<0.01	<0.01
TglH^{H231A}IA-Cys	0.65±0.01	0.02±0.01	<0.01	<0.01

All proteins were expressed in *E. coli* BL21 grown in LB medium supplemented with 1 mM ammonium ferrous sulfate. All the values are mean and standard deviation of the measured metal concentrations for three independent protein preparations.

Table S3. The recipe of minimal nutrient M9 medium (Related to STAR Methods)

Reagent	Content (Final concentration in 1L)
Na_2HPO_4	8.86 g
KH_2PO_4	3.9 g
NaCl	0.65 g
NH_4Cl	1.3 g
Glucose	5 %
YNB medium without Fe/Cu	0.65 %
**Amino acids**	
L-Met	50 mg
L-Leu	50 mg
L-Ile	50 mg
L-Val	50 mg
L-Lys	100 mg
L-Thr	100 mg
L-Phe	100 mg
ddH_2O	Make up the volume

YNB medium was from BD Difco. And the other regents were purchased from MilliporeSigma (Darmstadt Germany)

Table S4. The primer pairs for TglH and TglI mutants (Related to STAR Methods)

Mutants	Forward primer	Reverse primer
	**TglH**	
H71A	GCGTCCAATTACGGCTGCGATTTTGGTTTCAGTG	AGCCAGCAAGGTAAATTTTTGCCCTAGCC
H107A	TGTTTCTATGGGGATCAGTCCTGG	CGCGTTGGCCACCCAAGGGC
E151A	AATGCCGCCTACTACCTTGAATGC	CGCATGAGCCAGCGGCATGCC
D184A	CTGCACAACATTTACACCAACCACCTCAATCTGAAAGG	CGCAAGGTGAAGAAAAGTCCCTGAGCGCTGC
N187A	ATTTACACCAACCACCTCAATCTGAAAGG	CGCGTGCAGGTCAAGGTGAAGAAAAGTCC
H216A	GCGCTGGCCGGAGGAAGCTGG	CACCGAAATCACCTTGTCCAGC
D229A	GCGTGGCATGACGCCTGCGTG	GTGATACAGGCCGCCGTGC
H231A	GCGGACGCCTGCGTGCCC	CCAGTCGTGATACAGGCCGC
E258A	GCGTATCAGGGGCAGGCGCATC	AAGAATCACAGCAGAGGGCTGTG
N73A	CTGCTCGCGCATAGCGCCTATGGCTGCGATTTTG	CAAAATCGCAGCCATAGGCGCTATGCGCGAGCAG
N73D	CTGCTCGCGCATAGCGACTATGGCTGCGATTTTG	CAAAATCGCAGCCATAGTCGCTATGCGCGAGCAG
N73L	CTGCTCGCGCATAGCCTCTATGGCTGCGATTTTG	CAAAATCGCAGCCATAGAGGCTATGCGCGAGCAG
△L1	CTGCTGGACAACATCATCAAACCAAGTGCG	CGCACTTGGTTTGATGATGTTGTCCAGCAG
△L2	GCCAACCACTGCTTCAGCCCAATCCAGTTC	GAACTGGATTGGGCTGAAGCAGTGGTTGGC
△L3	CACGAAAATGCGGCGCGCGAAGCCGAATTTC	GAAATTCGGCTTCGCGCGCCGCATTTTCGTG
△L4	GAATACCAAGGCCAAGATGCCAGCGACGAAAGCATGATCG	TTCGTCGCTGGCATCTTGGCCTTGGTATTCGAGGATAACC
	**TglI**	
R183A	TGGCTGGCTCAGAAAGATCAGCTG	TTTCTGAGCCAGCCAACCACTCAG
K185A	CGTCAGGCAGATCAGCTGGGTCTG	CTGATCTGCCTGACGCAGCCAACC
Y203A	CTGATTGCTCTGCCGACGCTGCAAG	CGGCAGAGCAATCAGCACGTGTTC
K212A	CGCCATGCATTCACGCTGATCAATG	CGTGAATGCATGGCGGGCTTGCAG
△α1	CACAGCCAGGATCCGGATGAACGTCTGCAAG	CTTGCAGACGTTCATCCGGATCCTGGCTGTG
△α3	CTGGCTCAGTGGTTTCGCCCAGCAGTTCGATC	GATCGAACTGCTGGGCGAAACCACTGAGCCAG
△199-216	CGGAAAGCACCGAAAATGCGCAAGCCGCCCGCCTC	GGCGGCTTGCGCATTTTCGGTGCTTTCCGGCAGACG
△CTD	CTGTACCGCGGTCTGTAAAAGCTTGCGGCCGCATAATGC	GGCCGCAAGCTTTTACAGACCGCGGTACAGACGAAC
